# Supplementary material for: Interactome Mapping of eIF3A in a Colon Cancer and an Immortalized Embryonic Cell Line Using Proximity-Dependent Biotin Identification
Source: Cancers (Basel). 2021 Mar 14;13(6):1293. doi: 10.3390/cancers13061293 (PMC7999522; doi:10.3390/cancers13061293)

Supplementary Materials

Interactome mapping of eIF3A in a colon cancer and an immortalized embryonic cell line using proximity-dependent biotin identification

Diep-Khanh Vo, Alexander Engler, Darko Stoimenovski, Roland Hartig, Thilo Kaehne, Thomas Kalinski, Michael Naumann, Johannes Haybaeck and Norbert Nass

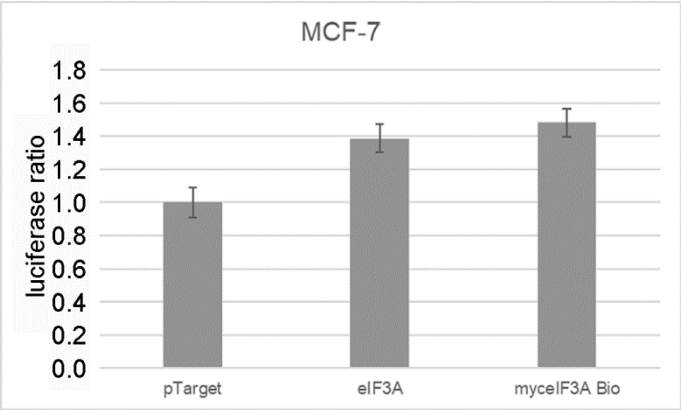

Figure S1: Effect of eIF3A and myc-BirA*-eIF3A over-expression on the polio-IRES to cap mediated translation ratio. The fusion protein acts similar as the native eIF3A, holding for a functional integration into the initiation complex. MCF-7 cells were transfected with a control vector (pTarget) and pTarget-based over expression vectors for native eIF3A and the birA-eIF3A fusion protein. Average of the luciferase ratio and standard deviation is shown. The differences to the empty-vector-control are significant with $p < 0.01$.

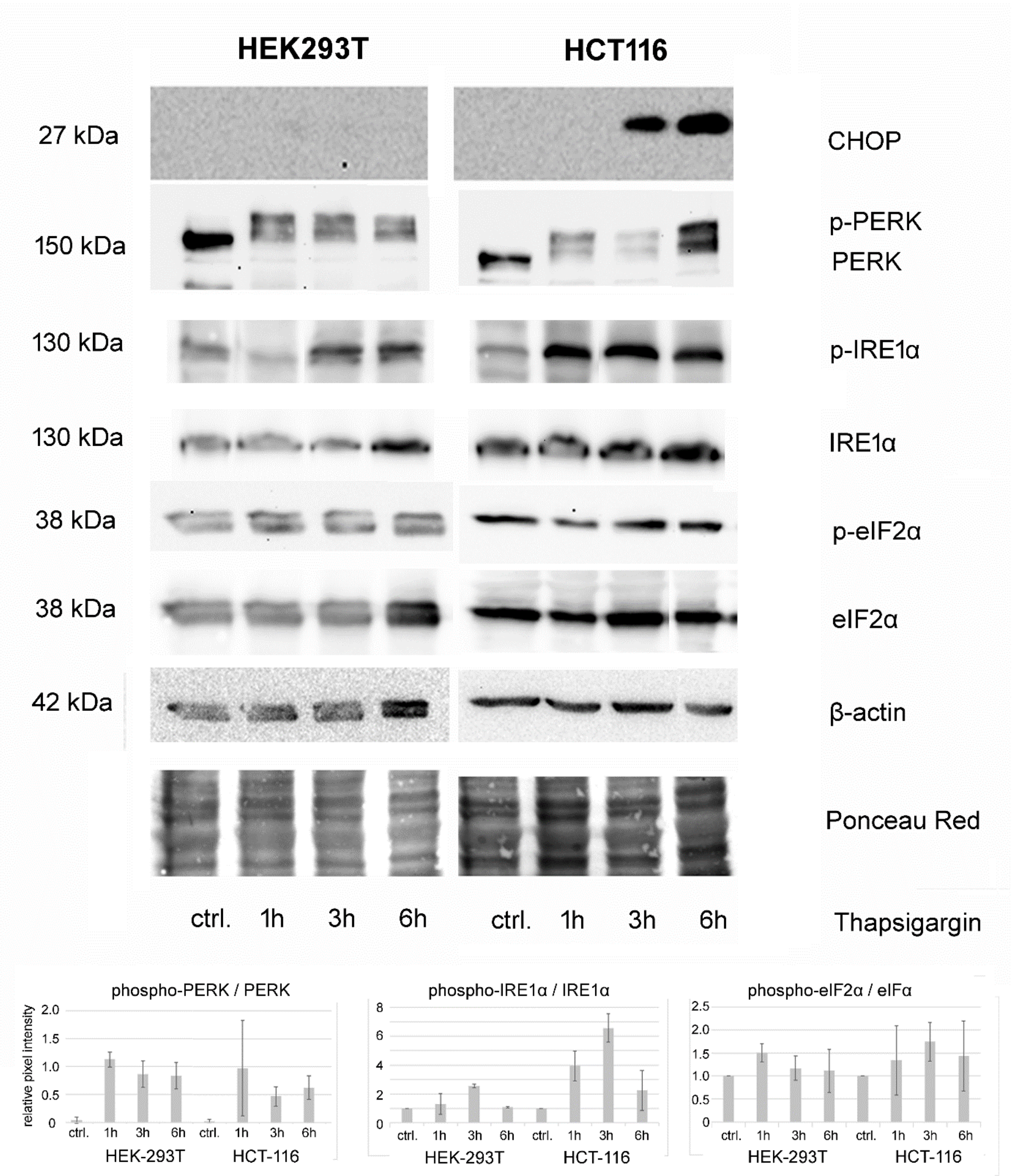

Figure S2: Signalling in HCT-116 and HEK-293-T cells upon incubation with thapsigargin. Cells were incubated with thapsigargin for the indicated time and proteins extracted and subjected to Western blotting as described in materials and methods (Vo et al 2019). Pixel intensity of the lanes was quantified using ImageJ and the results are shown in the lower panel.

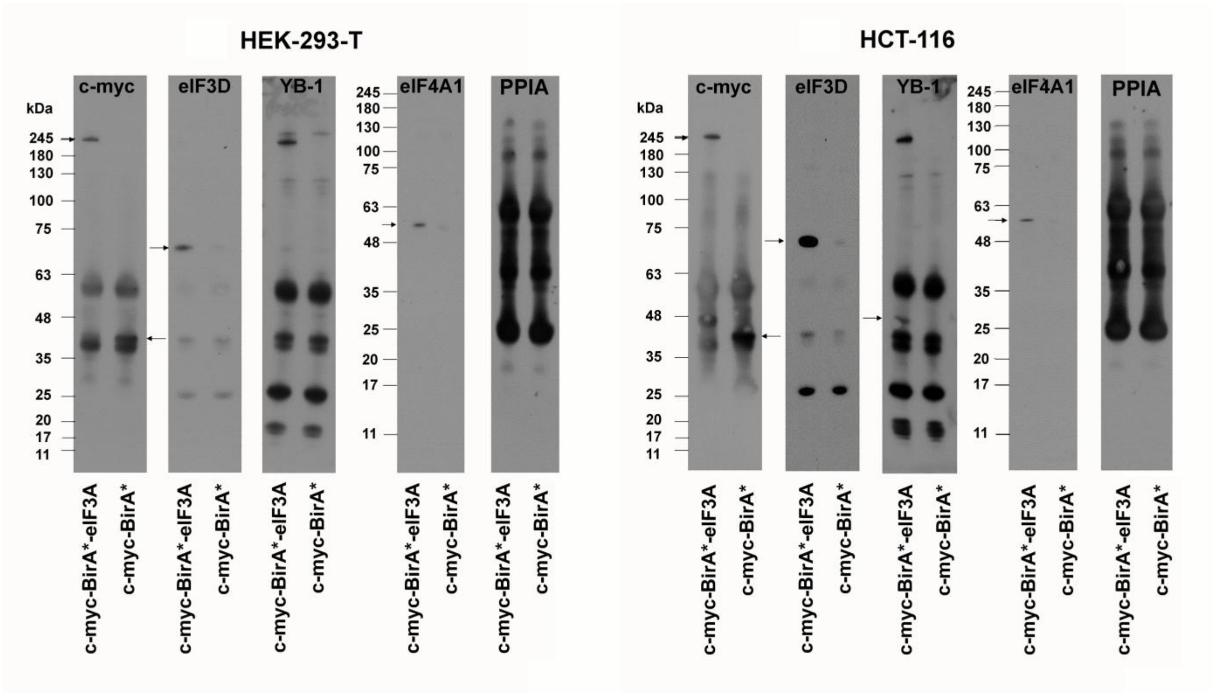

Figure S3: Co-immunoprecipitation assay. Western blot of the immunoprecipitated proteins probed for c-myc, eIF3D, eIF4A1, YB-1 and PPIA. Antibodies are indicated at the top of the X-ray films. Arrows mark the specific signals.

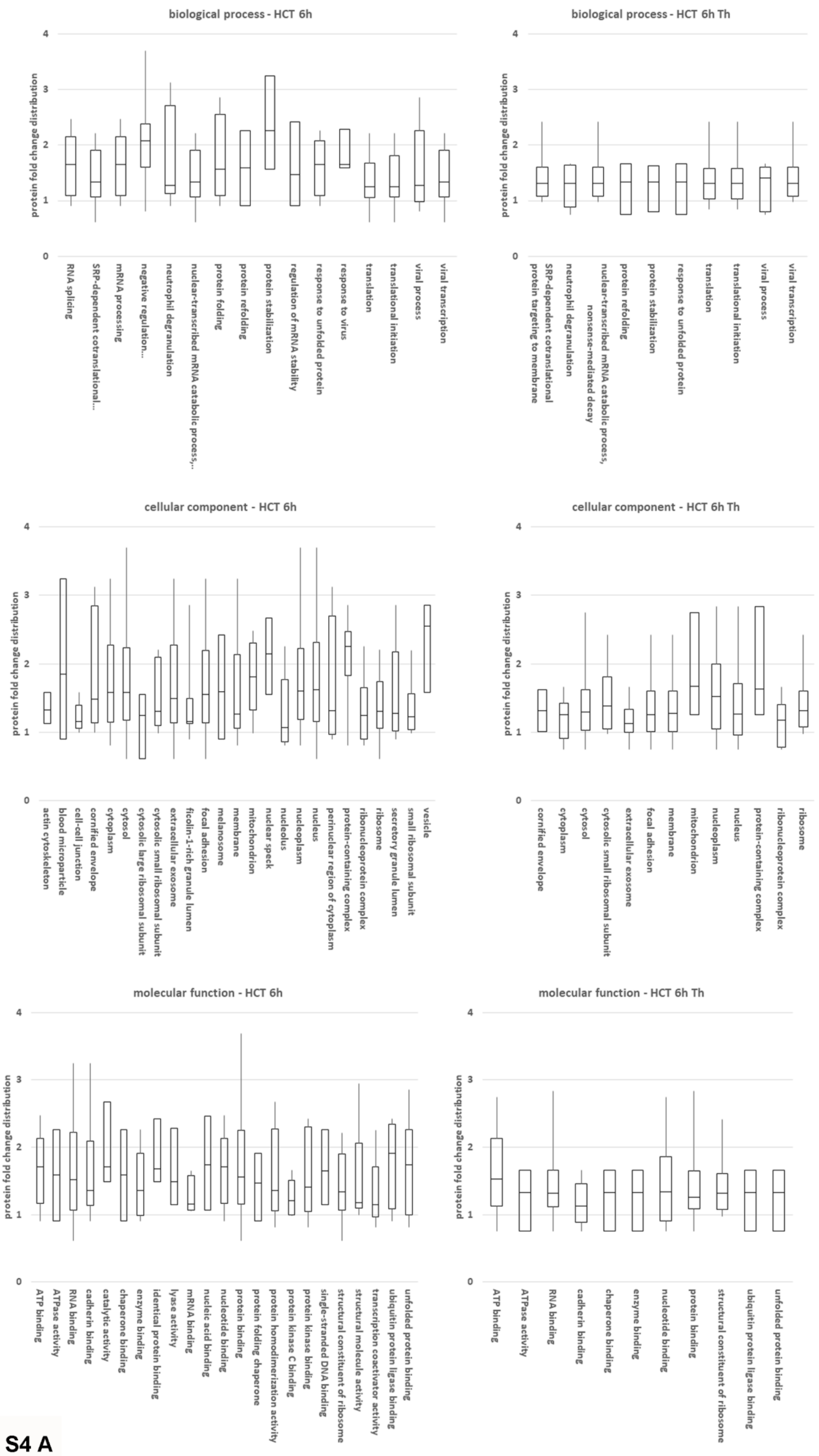
biological process - HCT 6h
protein fold change distribution
0
1
2
3
4
RNA splicing
SRP-dependent cotranslational...
mRNA processing
negative regulation...
neutrophil degranulation
nuclear-transcribed mRNA catabolic process,...
protein folding
protein refolding
protein stabilization
regulation of mRNA stability
response to unfolded protein
response to virus
translation
translational initiation
viral process
viral transcription
biological process - HCT 6h Th
protein fold change distribution
protein targeting to membrane
SRP-dependent cotranslational
neutrophil degranulation
nonsense-mediated decay
nuclear-transcribed mRNA catabolic process,
protein refolding
protein stabilization
response to unfolded protein
translation
translational initiation
viral process
viral transcription
cellular component - HCT 6h
protein fold change distribution
actin cytoskeleton
blood microparticle
cell-cell junction
cornified envelope
cytoplasm
cytosol
cytosolic large ribosomal subunit
cytosolic small ribosomal subunit
extracellular exosome
ficolin-1-rich granule lumen
focal adhesion
melanosome
membrane
mitochondrion
nuclear speck
nucleolus
nucleoplasm
nucleus
perinuclear region of cytoplasm
protein-containing complex
ribonucleoprotein complex
ribosome
secretory granule lumen
small ribosomal subunit
vesicle
cellular component - HCT 6h Th
protein fold change distribution
cornified envelope
cytoplasm
cytosol
cytosolic small ribosomal subunit
extracellular exosome
focal adhesion
membrane
mitochondrion
nucleoplasm
nucleus
protein-containing complex
ribonucleoprotein complex
ribosome
molecular function - HCT 6h
protein fold change distribution
ATP binding
ATPase activity
RNA binding
cadherin binding
catalytic activity
chaperone binding
enzyme binding
identical protein binding
lyase activity
mRNA binding
nucleic acid binding
nucleotide binding
protein binding
protein folding chaperone
protein homodimerization activity
protein kinase C binding
protein kinase binding
single-stranded DNA binding
structural constituent of ribosome
structural molecule activity
transcription coactivator activity
ubiquitin protein ligase binding
unfolded protein binding
molecular function - HCT 6h Th
protein fold change distribution
ATP binding
ATPase activity
RNA binding
cadherin binding
chaperone binding
enzyme binding
nucleotide binding
protein binding
structural constituent of ribosome
ubiquitin protein ligase binding
unfolded protein binding

S4 A

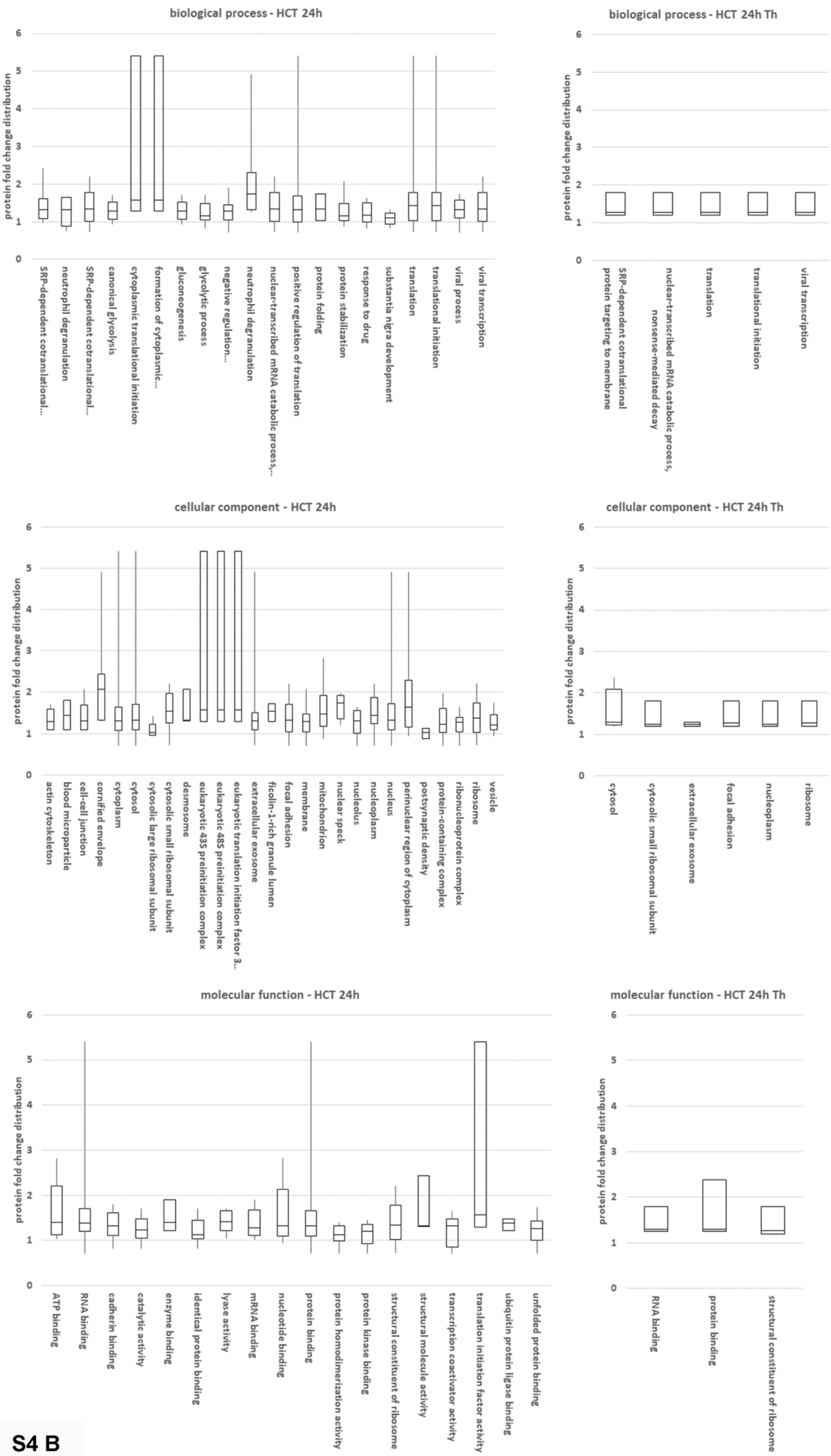

biological process - HCT 24h
protein fold change distribution
0
1
2
3
4
5
6
SRP-dependent cotranslational...
neutrophil degranulation
SRP-dependent cotranslational...
canonical glycolysis
cytoplasmic translational initiation
formation of cytoplasmic...
gluconeogenesis
glycolytic process
negative regulation...
neutrophil degranulation
nuclear-transcribed mRNA catabolic process,...
positive regulation of translation
protein folding
protein stabilization
response to drug
substantia nigra development
translation
translational initiation
viral process
viral transcription
biological process - HCT 24h Th
SRP-dependent cotranslational protein targeting to membrane
nuclear-transcribed mRNA catabolic process, nonsense-mediated decay
translation
translational initiation
viral transcription
cellular component - HCT 24h
actin cytoskeleton
blood microparticle
cell-cell junction
cornified envelope
cytoplasm
cytosol
cytosolic large ribosomal subunit
cytosolic small ribosomal subunit
desmosome
eukaryotic 43S preinitiation complex
eukaryotic 48S preinitiation complex
eukaryotic translation initiation factor 3...
extracellular exosome
ficolin-1-rich granule lumen
focal adhesion
membrane
mitochondrion
nuclear speck
nucleolus
nucleoplasm
nucleus
perinuclear region of cytoplasm
postsynaptic density
protein-containing complex
ribonucleoprotein complex
ribosome
vesicle
cellular component - HCT 24h Th
cytosol
cytosolic small ribosomal subunit
extracellular exosome
focal adhesion
nucleoplasm
ribosome
molecular function - HCT 24h
ATP binding
RNA binding
cadherin binding
catalytic activity
enzyme binding
identical protein binding
lyase activity
mRNA binding
nucleotide binding
protein binding
protein homodimerization activity
protein kinase binding
structural constituent of ribosome
structural molecule activity
transcription coactivator activity
translation initiation factor activity
ubiquitin protein ligase binding
unfolded protein binding
molecular function - HCT 24h Th
RNA binding
protein binding
structural constituent of ribosome

S4 B

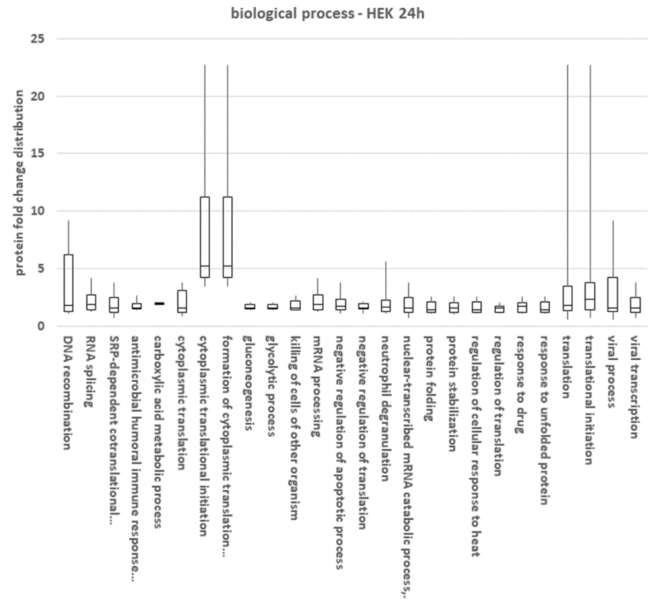

No significant hit for biological process
HEK 24h

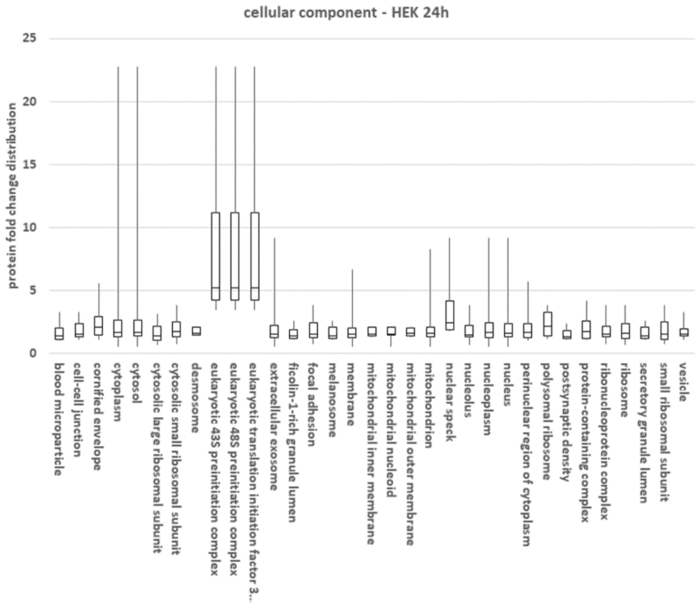

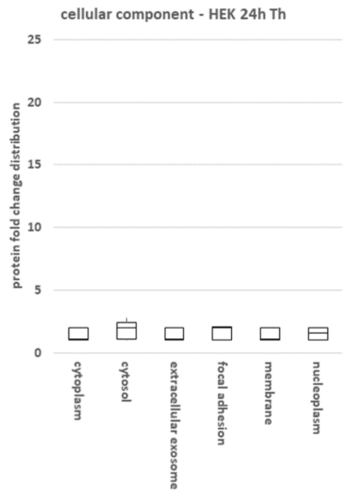

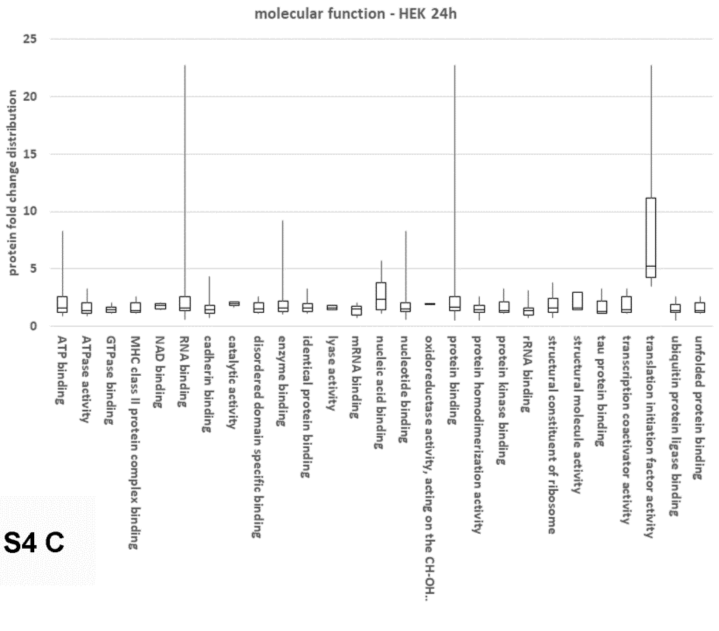

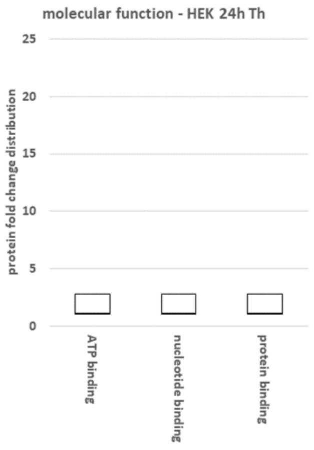

S4 C

Figure S4: GO term enrichment analysis using Genecodis. Mean interaction scores for the significant GO terms are shown as box plots for HCT-116 at 6h (A) and 24h (B) and HEK-293T at 24h (C). Only significant GO terms for cellular component, molecular function and biological process are shown.

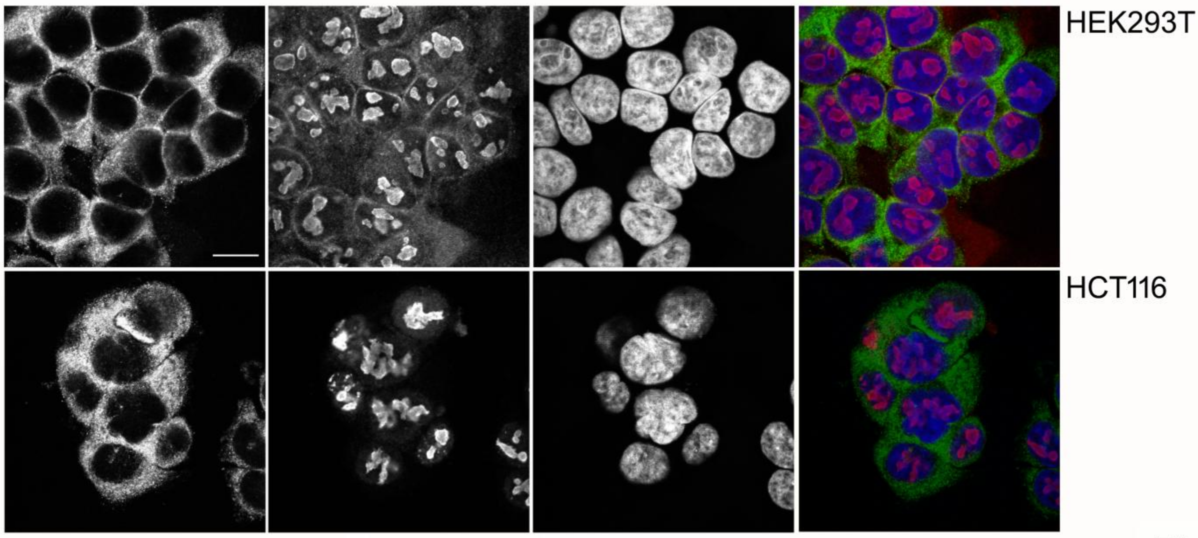
HEK293T
HCT116
eIF3a
nucleophosmin
DAPI
combined

5A

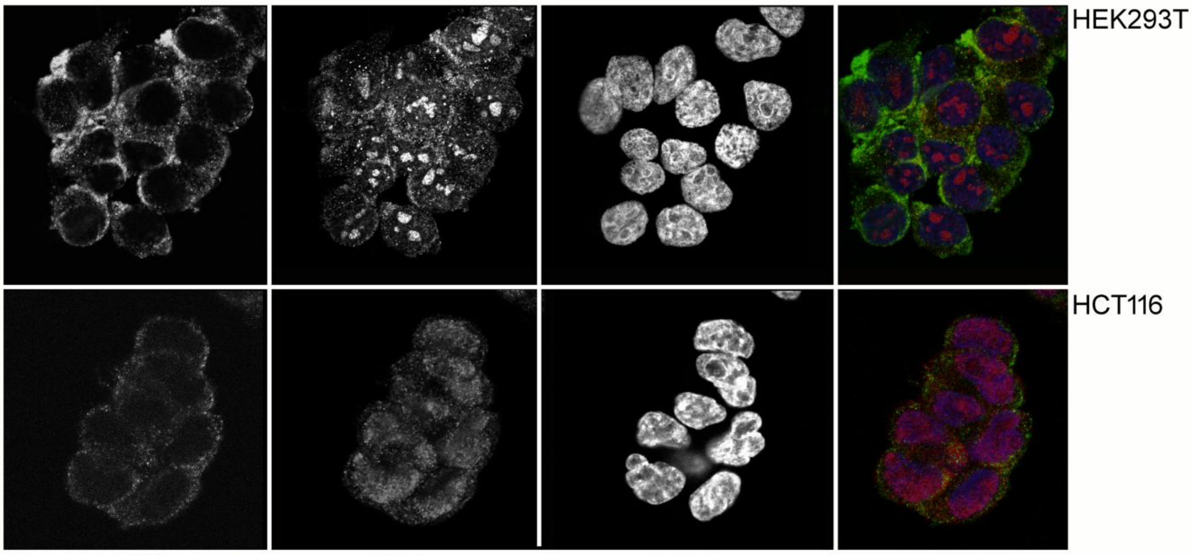
HEK293T
HCT116
eIF3A
PPIA
DAPI
combined

5B

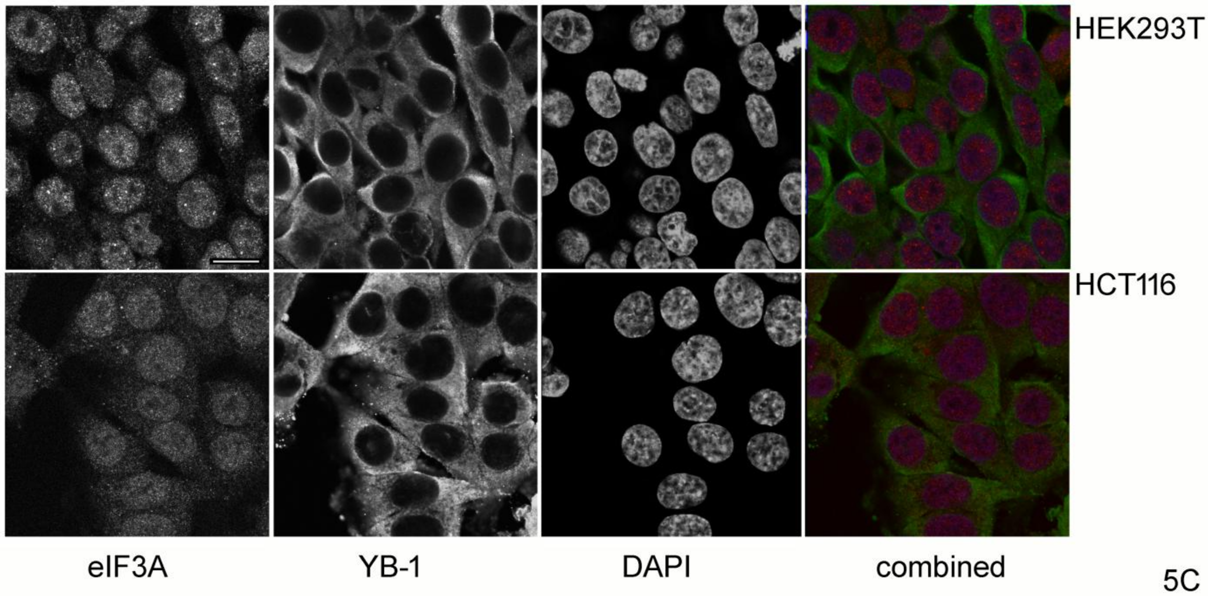
HEK293T
HCT116
eIF3A
YB-1
DAPI
combined

5C

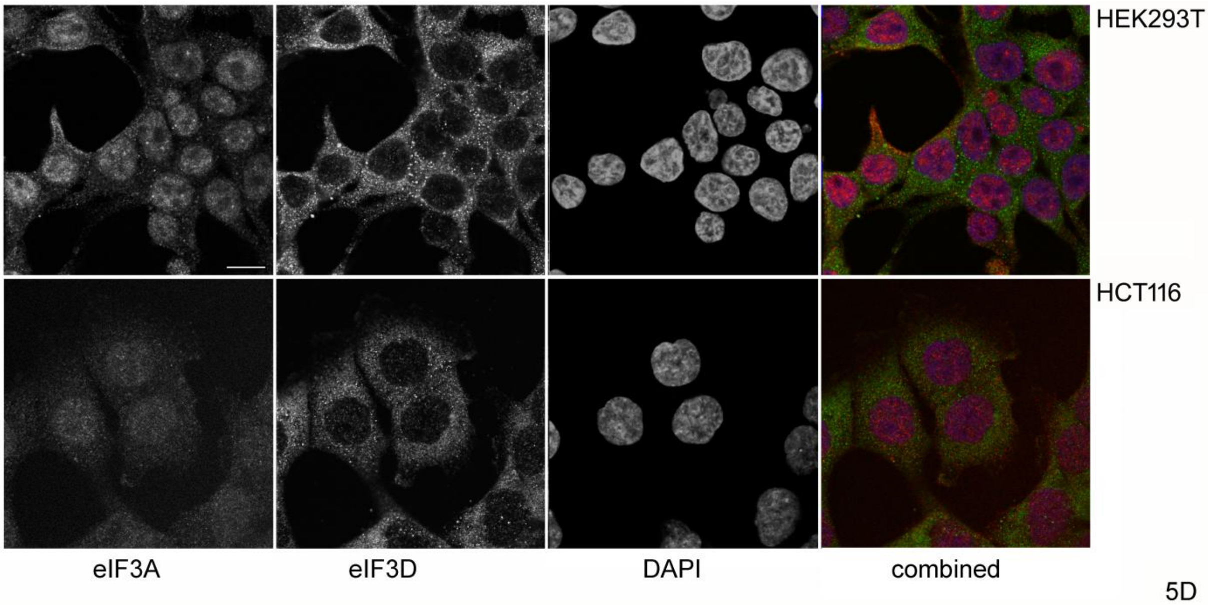
HEK293T
HCT116
eIF3A
eIF3D
DAPI
combined

5D

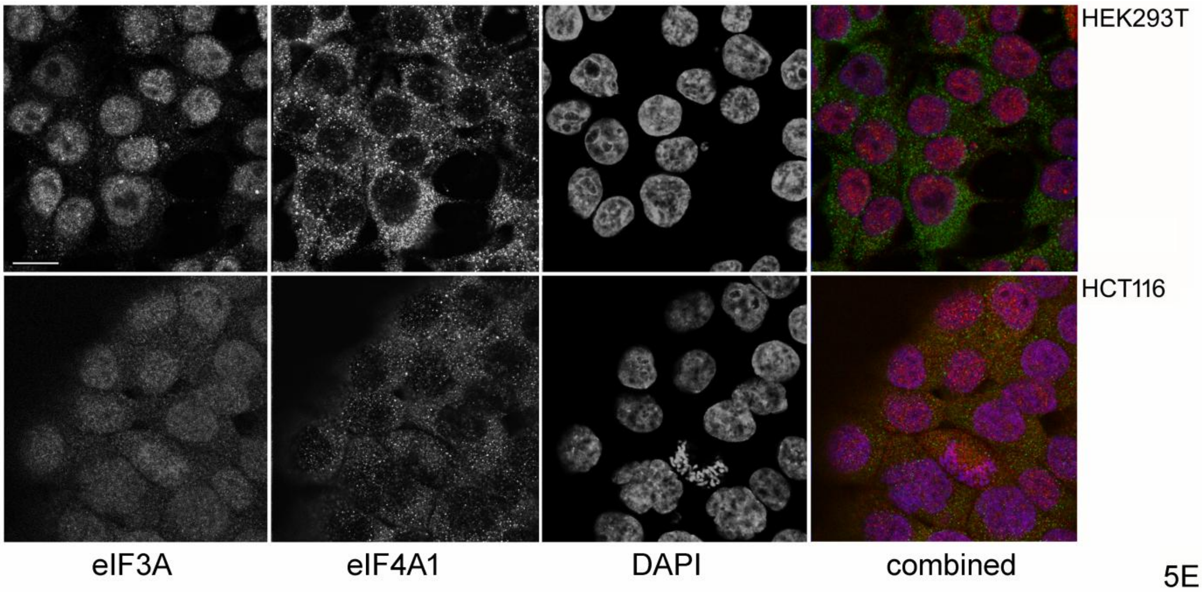
HEK293T
HCT116
eIF3A
eIF4A1
DAPI
combined

5E

Figure S5: Co-localization of eIF3A with nucleophosmin (A), cyclophilin (PPIA, B), YB-1 (C), eIF3D (D) and eIF4A (E) in HCT-116 and HEK-293-T cells under normal growth conditions. Note that in A and B a monoclonal rabbit antibody has been used for eIF3A which was detected with DyLight 488 labelled secondary antibody (green) and the second antigen was detected with monoclonal mouse antibodies using a Dylight 549 labelled secondary antibody (red). In C, D and E, eIF3A was detected with a monoclonal mouse antibody and detected by a Dylight 549 labelled secondary antibody (red) and the second antigen was detected with a rabbit polyclonal serum and Dylight 488 (green). The scale bar represents 50 μm.

Table S1: Ratio of m^7G-cap to Polio-IRES dependent translation. Cells were transfected with a vector encoding *Renilla* luciferase and *Photinus* luciferase on one transcript separated by the Polio- IRES sequence (pcDNA3-RLUC-POLIRES-FLUC). A dual luciferase assay was performed and the ratio of *Photinus* to *Renilla* luciferase determined. For better comparison of the three independent experiments with 8 replicas each, the data for the HEK-293T cell-line in individual experiments was set to 1. Geometric average +/- standard error are shown. Significance was calculated by one way ANOVA.

**Cell-line**	**HEK-293T**	**HCT-116**	**Significance**
Photinus (IRES) to *Renilla* (cap) luciferase ratio	1.00 +/- 0.01	1.12 +/- 0.03	P= 0.04

Uncropped blots included in Figure 1: Biotin.

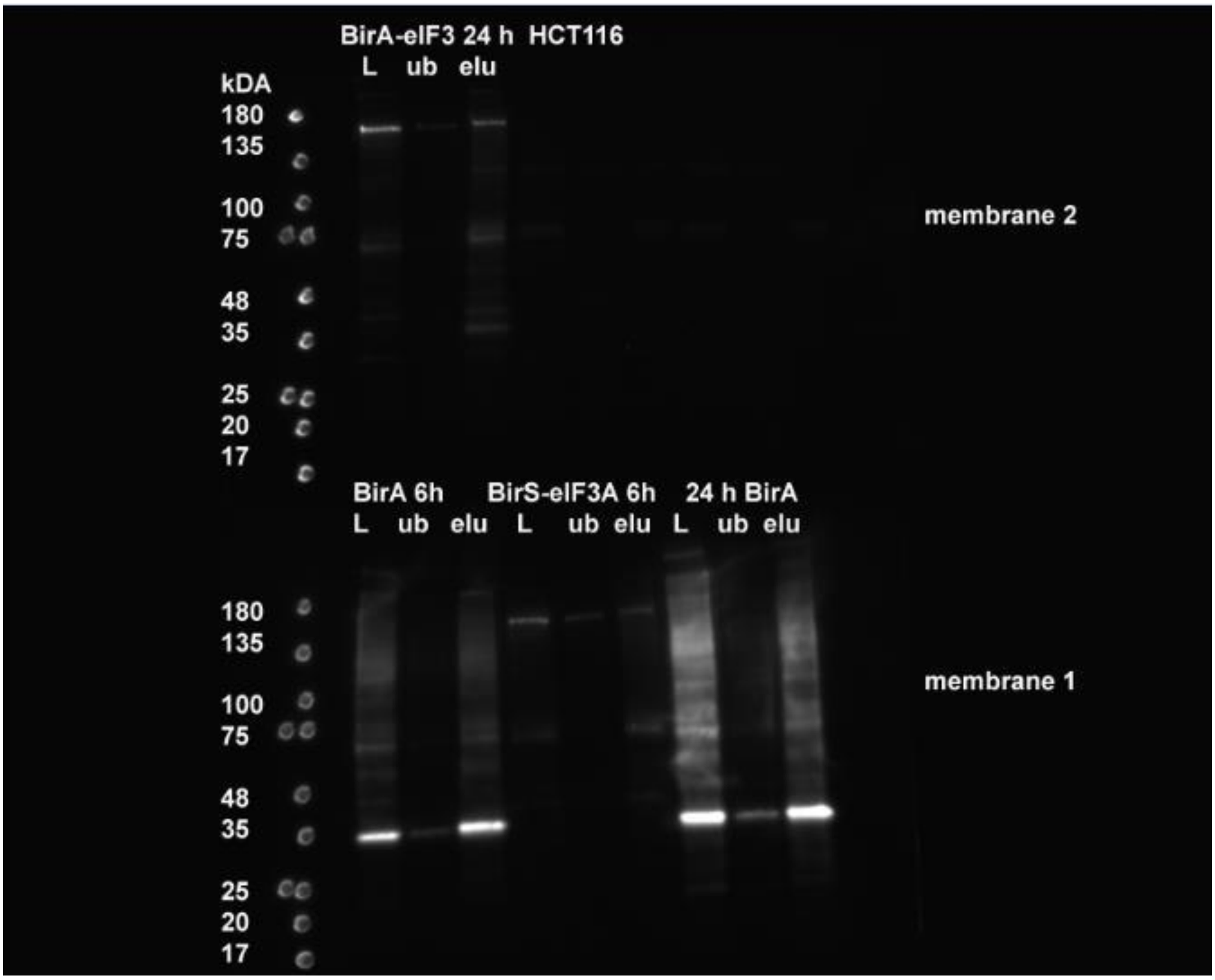

Original information of Figure 1: eIF3A.

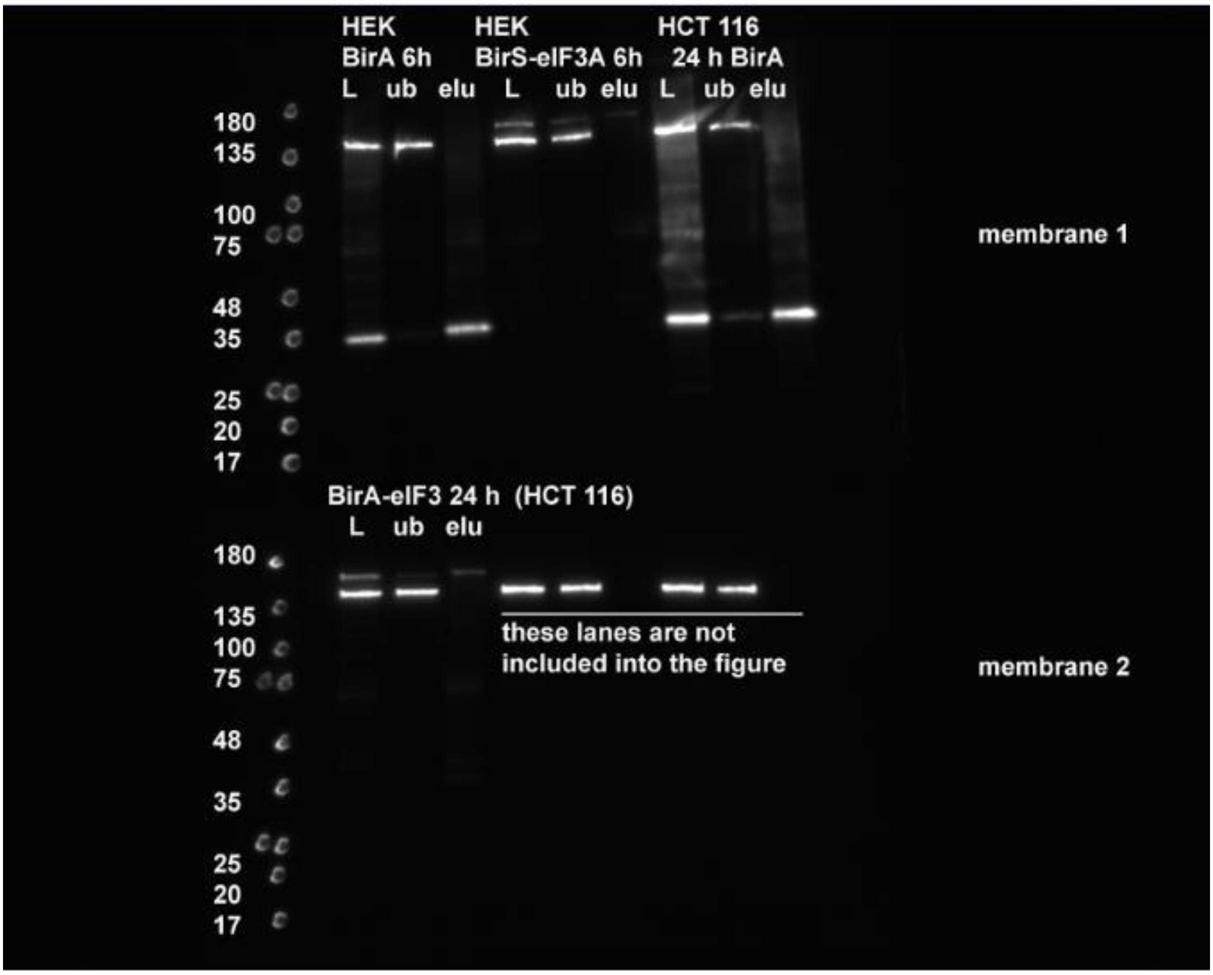

Uncropped blots for Fig.S1: Ponceau Red exp1

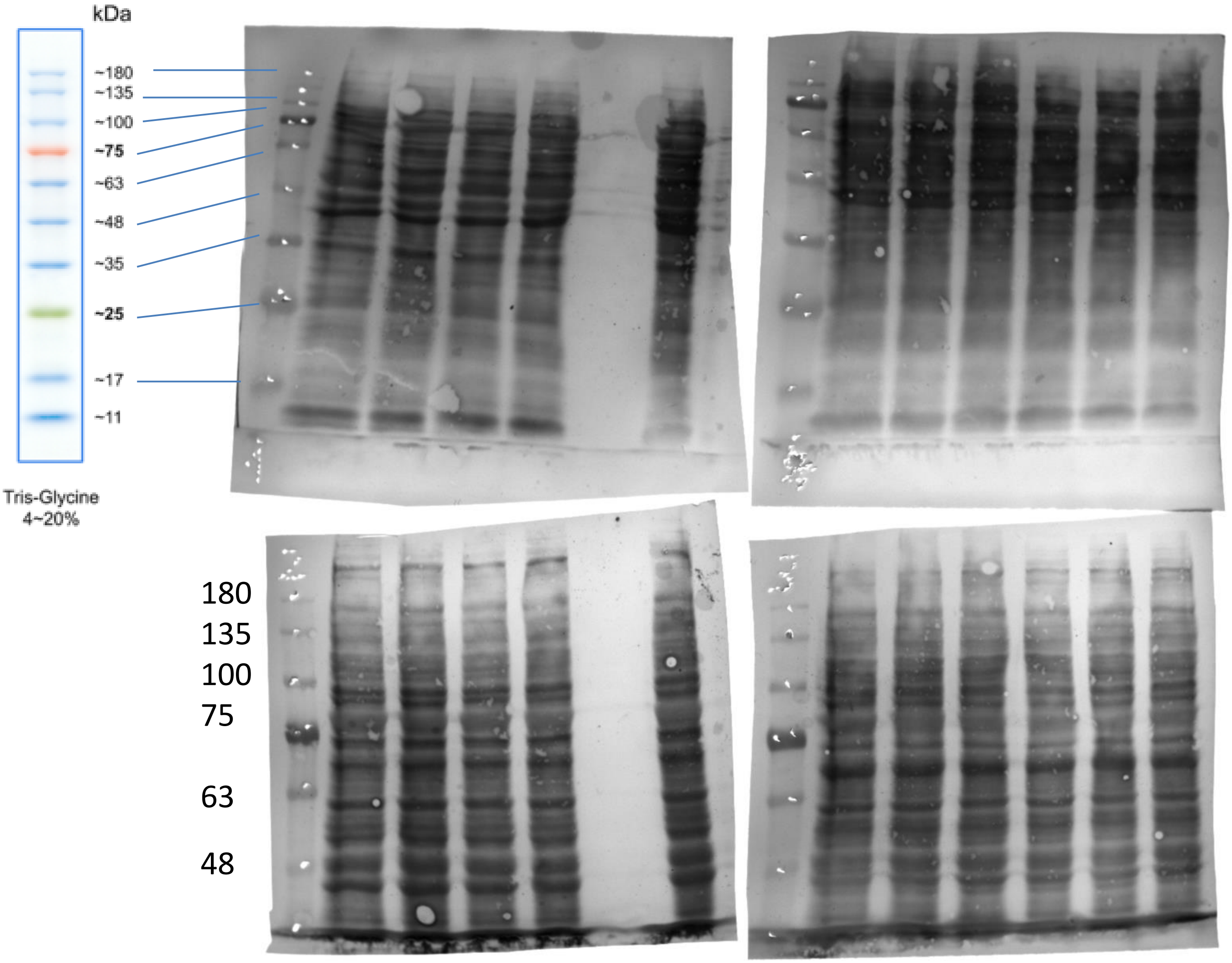

Loading: upper gel 12.5%, lower gel 7.5%

Left: HCT: Marker ctrl 1h 3h 3h 6h 6h HEK: ctrl 1h 3h 3h 6h 6h

Green labelled lanes are included in Fig.S1

Uncropped blots for Fig.S1 P-eIF2alpha Exp 1: 12.5% gel

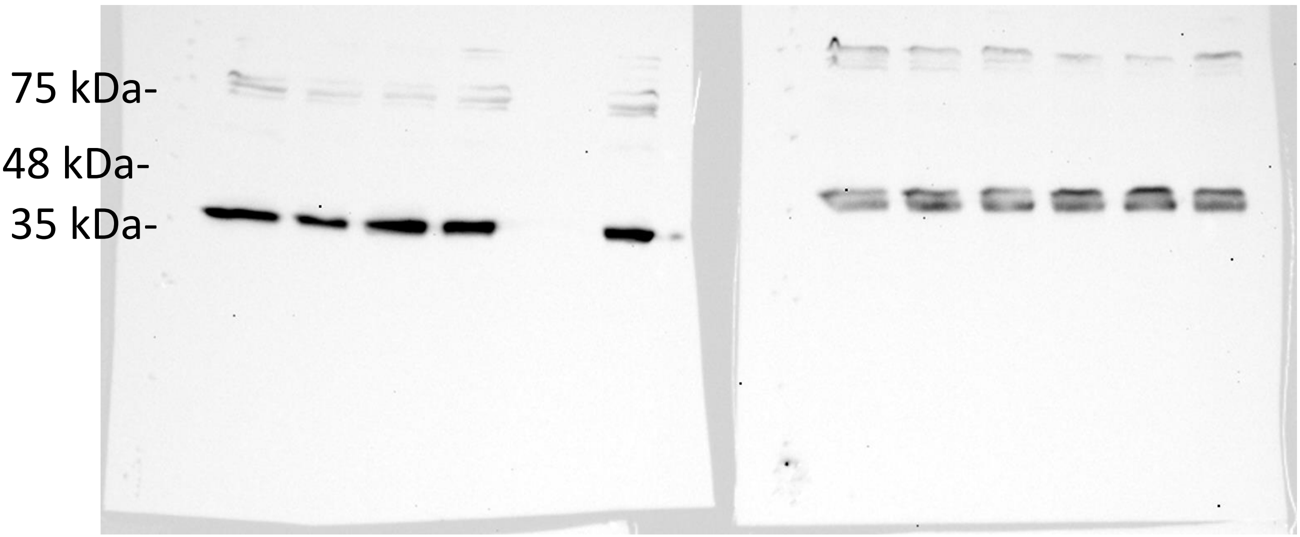

Left: HCT: Marker ctrl 1h 3h 3h 6h 6h Right: HEK: ctrl 1h 3h 3h 6h 6h

Uncropped blots for Fig.S1: eIF2alpha Exp 1 12.5 % gel:

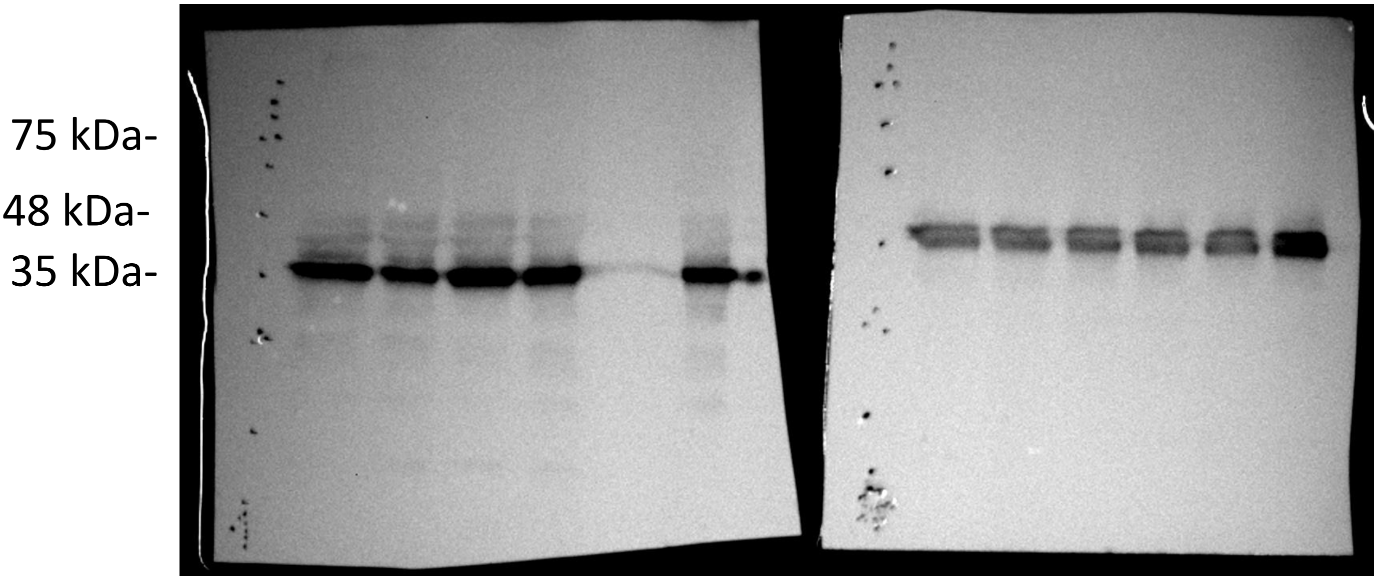

Left: HCT: Marker ctrl 1h 3h 3h 6h 6h Right: HEK: ctrl 1h 3h 3h 6h 6h

Uncropped blots for Fig.S1: PERK exp 1, 7.5% gel

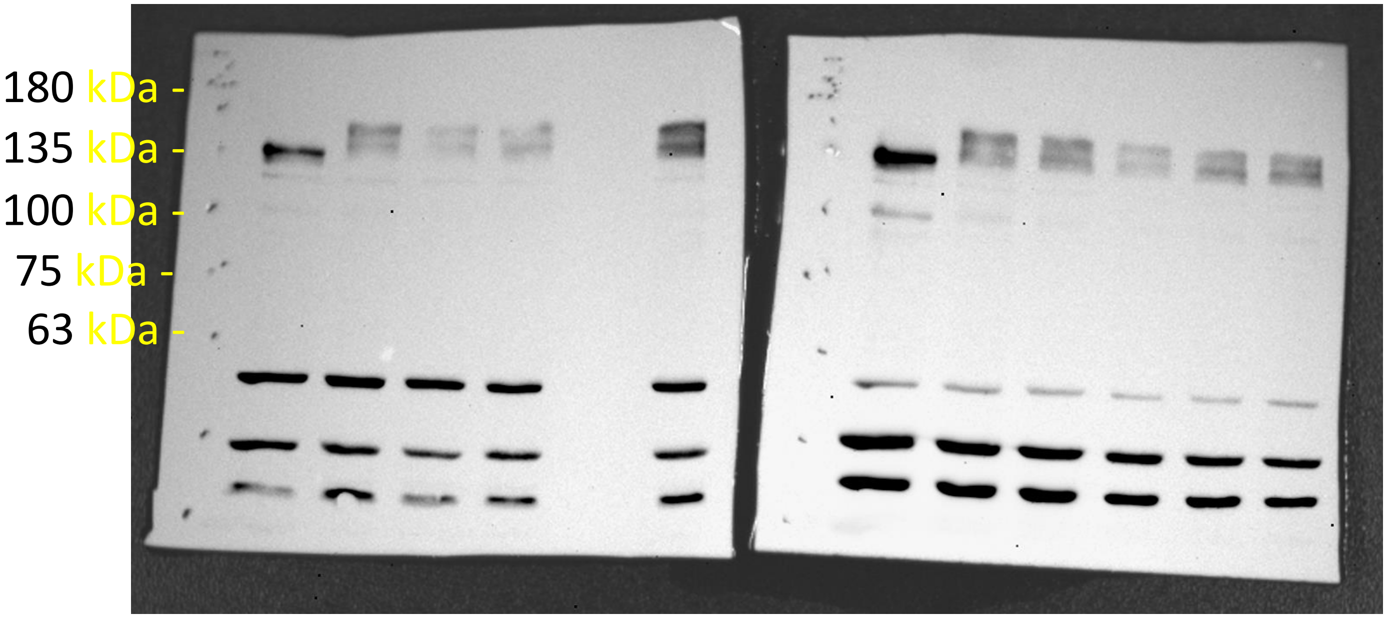

Left: HCT: Marker ctrl 1h 3h 3h 6h 6h Right: HEK: ctrl 1h 3h 3h 6h 6h

Uncropped blots for Fig.S1: ß-actin Exp 1, 12.5% gel

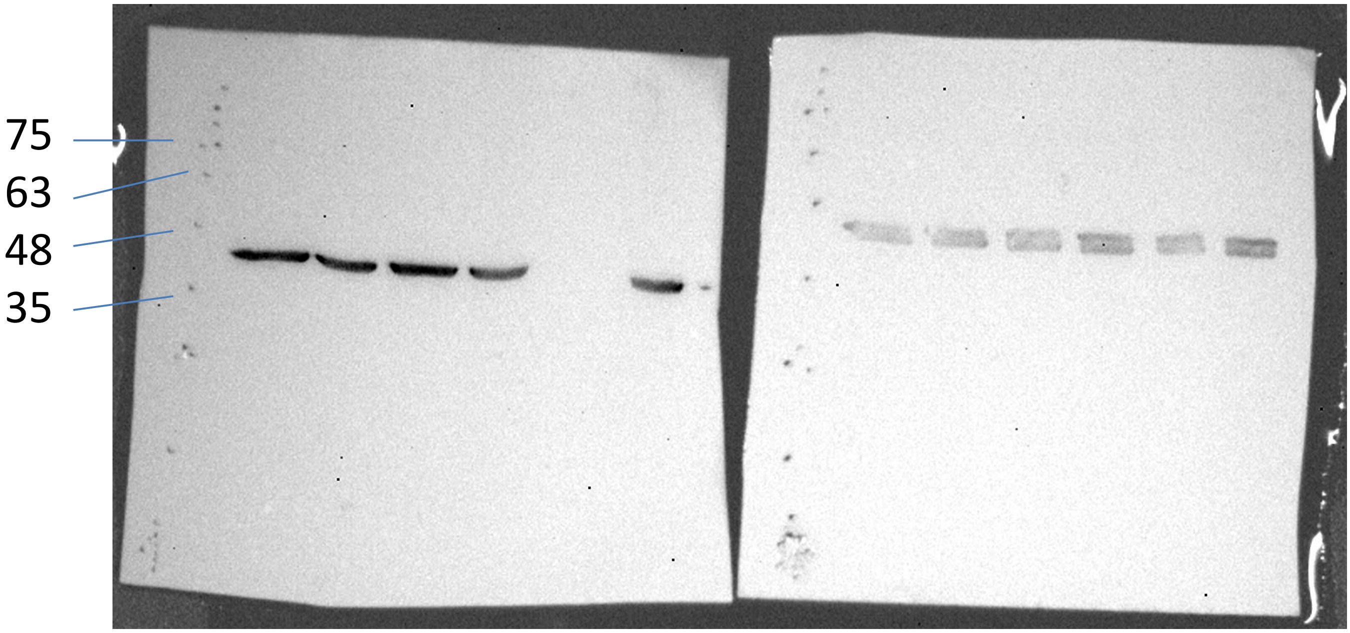

Left: HCT: Marker ctrl 1h 3h 3h 6h 6h Right: HEK: ctrl 1h 3h 3h 6h 6h

Uncropped blots for Fig. S1: Ponceau RedPonceau Red Exp. 2

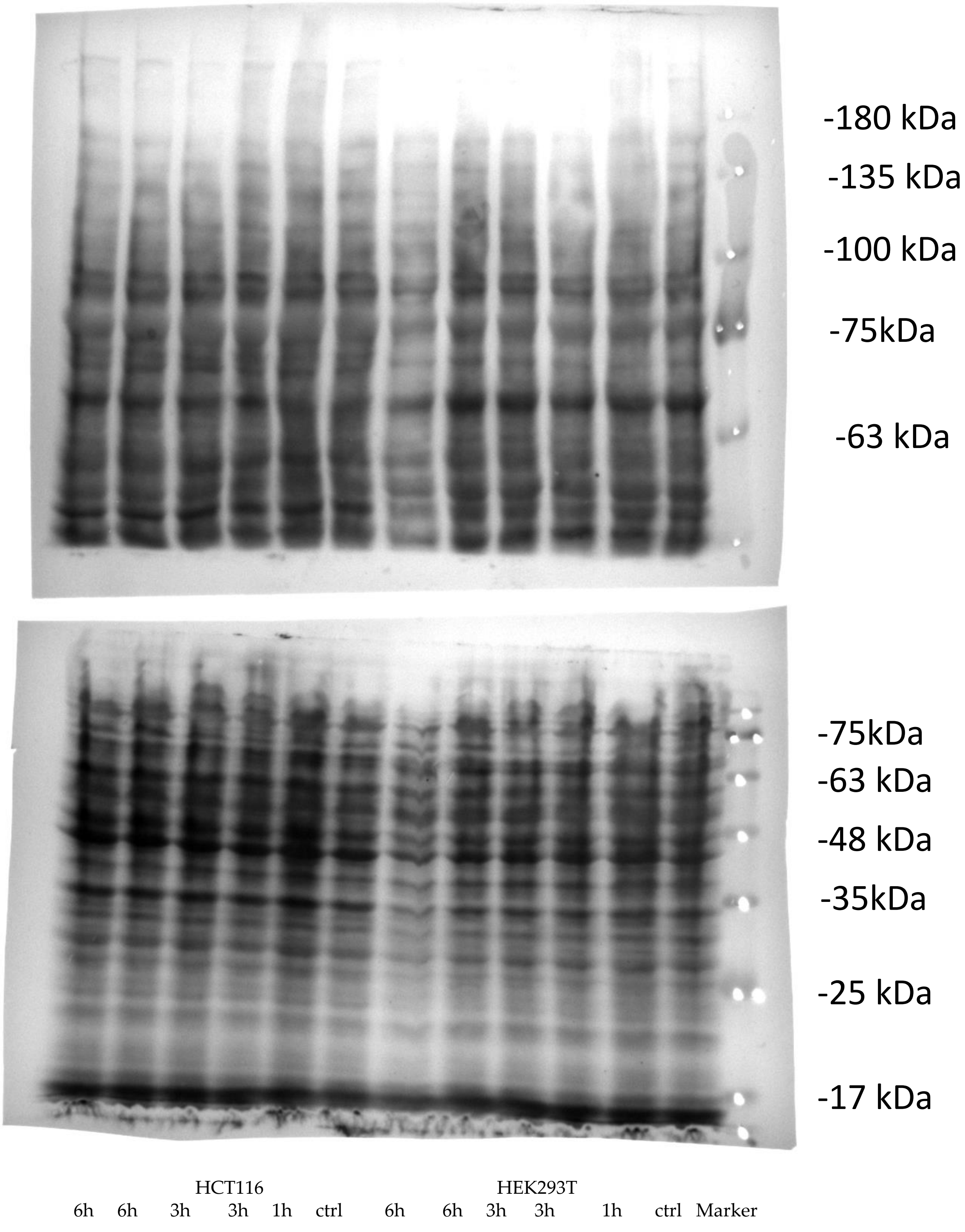

Uncropped blot for Fig. S1: CHOP, exp 2, 12.5 % gel

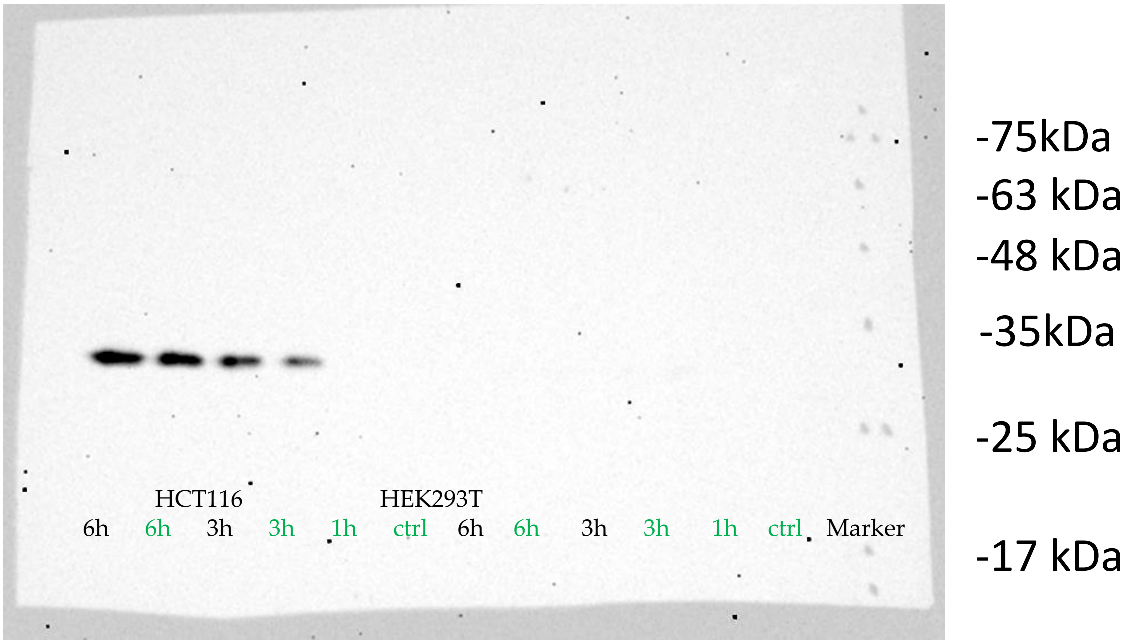

P-IRE1alpha Exp 2 gel 2

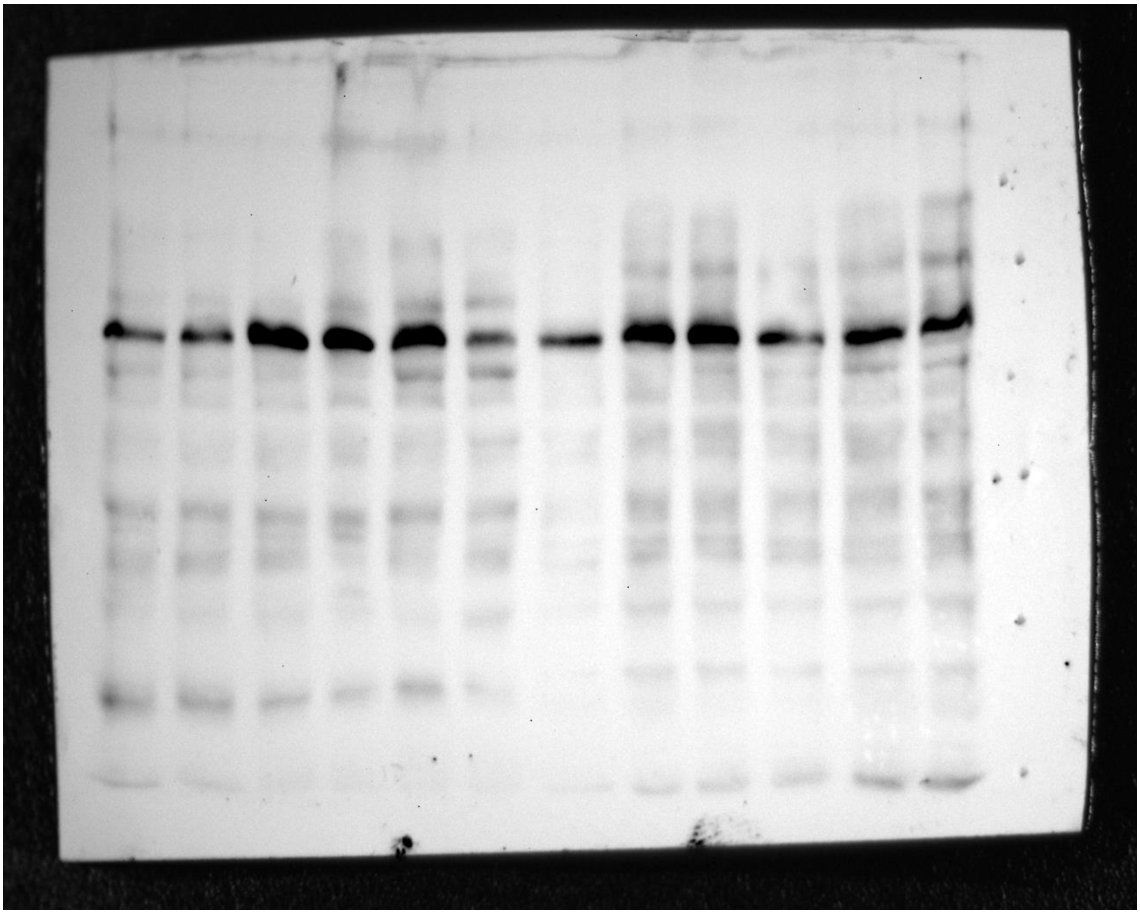

-180 kDa
-135 kDa
-100 kDa
-75kDa
-63 kDa

HCT116 HEK293T

6h 6h 3h 3h 1h ctrl 6h 6h 3h 3h 1h ctrl Marker

Uncropped blots for Fig. S1:pIRE1alpha Exp 2 Gel 4

180 kDa -
135 kDa -
100 kDa -
75kDa -
63 kDa -

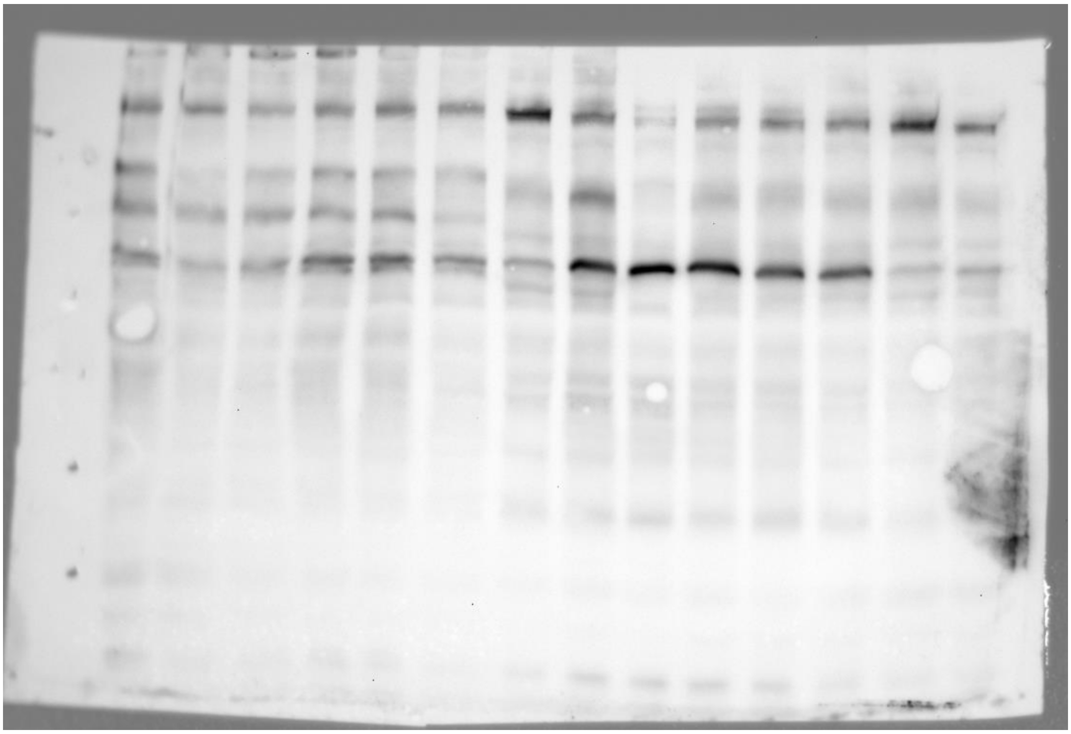

Loading: : M, HEK HCT

ctrl 1h, 3h, 3h, 6h, 6h, : ctrl, 1h, 3h, 3h, 6h, 6h, ctrl, ctrl

Uncropped blots for Fig. S1: IRE1alpha Exp 2 Gel 3

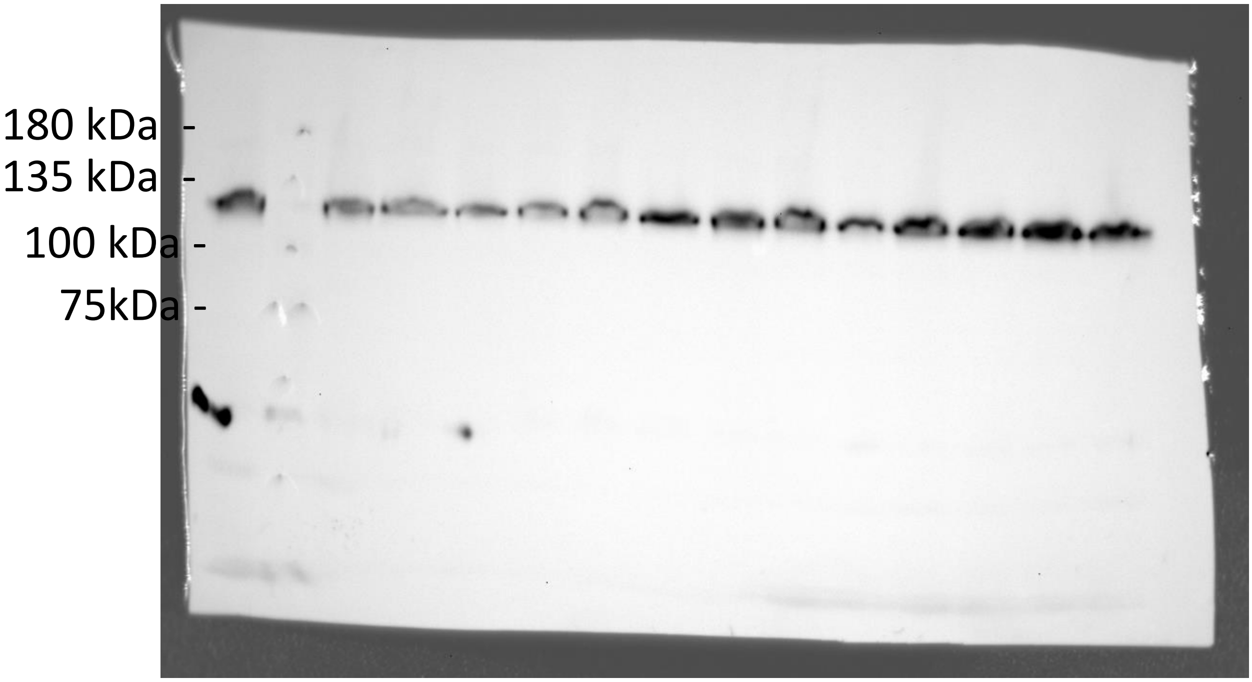

Loading: HEK HCT

ctrl, M, ctrl, 1h, 3h, 3h, 6h, 6h, : ctrl, 1h, 3h, 3h, 6h, 6h, ctrl

Uncropped blots for Fig. S1: Actin, Exp 2, 12.5% gel

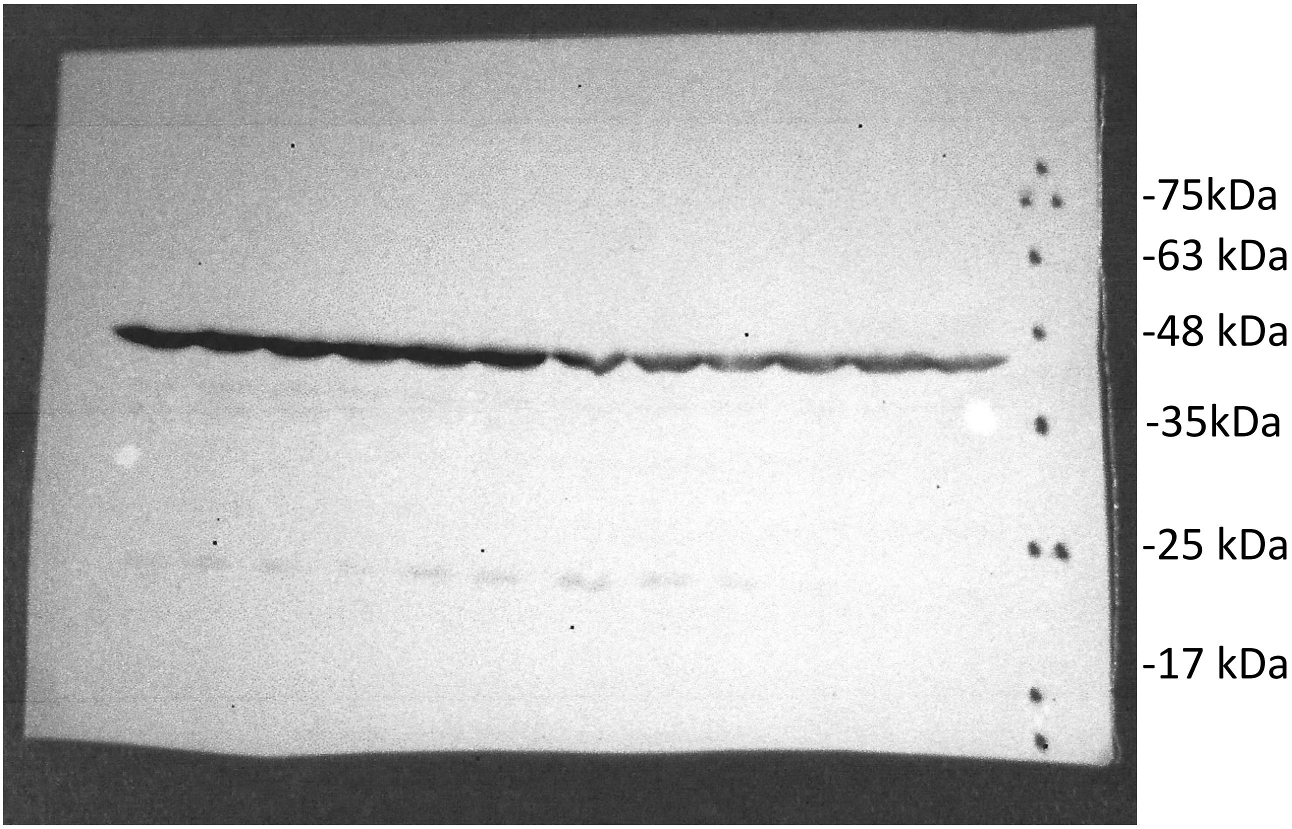

HCT116 HEK293T

6h 6h 3h 3h 1h ctrl 6h 6h 3h 3h 1h ctrl Marker

Uncropped blots for Fig. S1: P-EIF2-alpha, Exp 2, 12.5 % gel

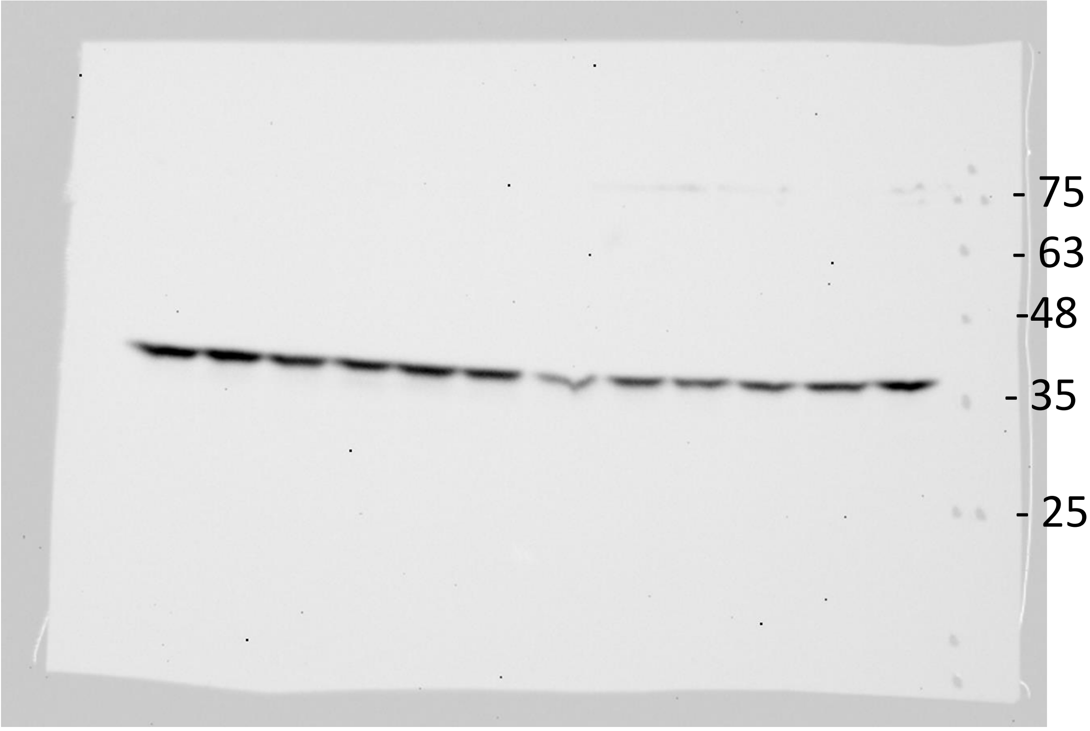

HCT116 HEK293T

6h 6h 3h 3h 1h ctrl. 6h 6h 3h 3h 1h ctrl. Marker

Uncropped blots for FigS1: eIF2-alpha

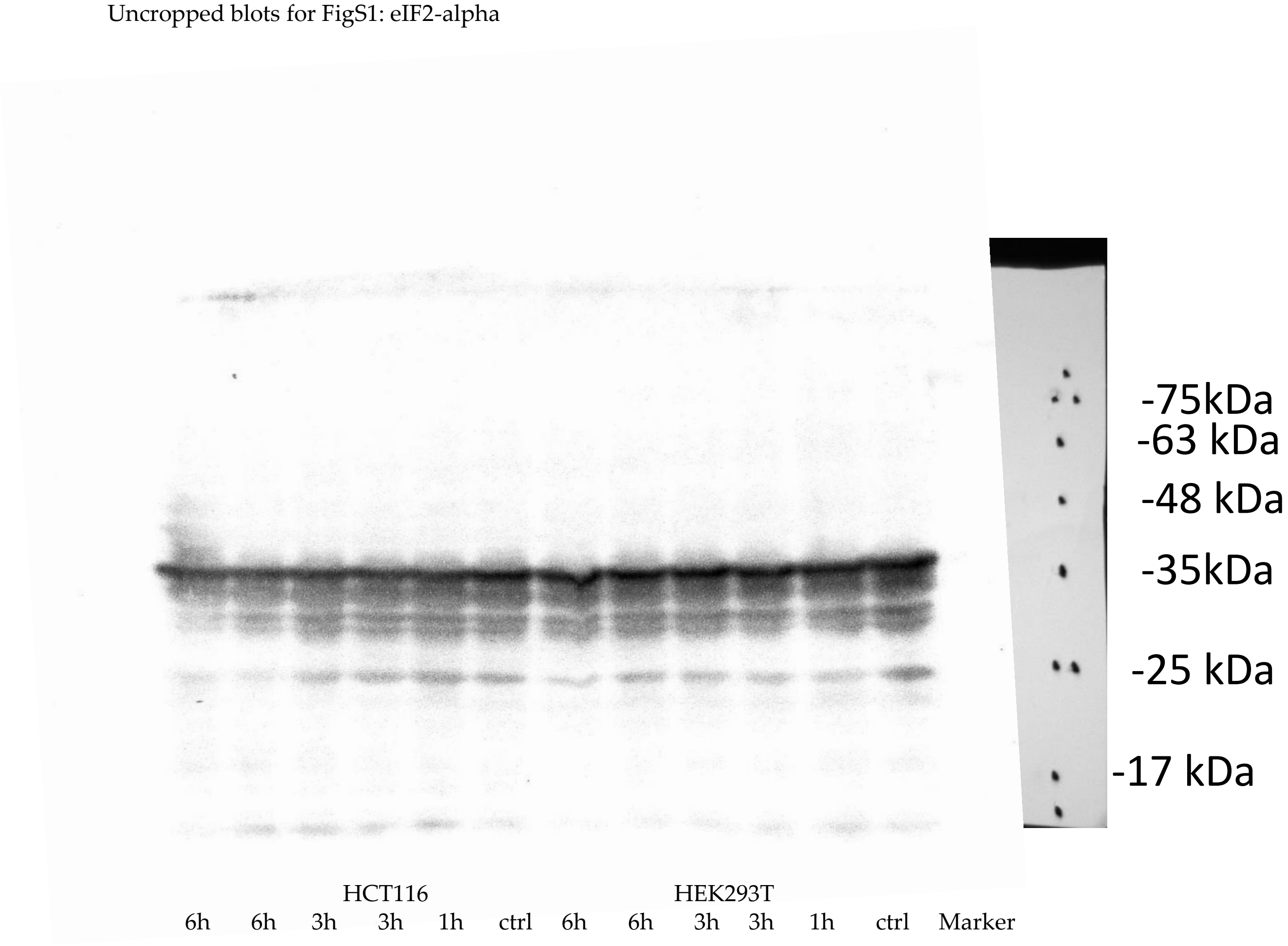

Supplement: Supplementary file 1 [file cancers-13-01293-s001.zip › supplementary materials.pdf]
