# Supplementary material for: Interactome Mapping of eIF3A in a Colon Cancer and an Immortalized Embryonic Cell Line Using Proximity-Dependent Biotin Identification
Source: Cancers (Basel). 2021 Mar 14;13(6):1293. doi: 10.3390/cancers13061293 (PMC7999522; doi:10.3390/cancers13061293)
Supplement: Supplementary file 1 [file cancers-13-01293-s001.zip › Table S1.docx]

Supplement table 1: Ratio of m^7^G-cap to Polio-IRES dependent translation. Cells were transfected with a vector encoding *Renilla* luciferase and *Photinus* luciferase on one transcript separated by the Polio- IRES sequence (pcDNA3-RLUC-POLIRES-FLUC). A dual luciferase assay was performed and the ratio of *Photinus* to *Renilla* luciferase determined. For better comparison of the three independent experiments with 8 replicas each, the data for the HEK-293T cell-line in individual experiments was set to 1. Geometric average +/- standard error are shown. Significance was calculated by one way ANOVA.

| **Cell-line** | **HEK-293T** | **HCT-116** | **Significance** |
| --- | --- | --- | --- |
| *Photinus* (IRES) to *Renilla* (cap) luciferase ratio | 1.00 +/- 0.01 | 1.12 +/- 0.03 | P= 0.04 |
