# Supplementary figures and images for: Interactome Mapping of eIF3A in a Colon Cancer and an Immortalized Embryonic Cell Line Using Proximity-Dependent Biotin Identification
Source: Cancers (Basel). 2021 Mar 14;13(6):1293. doi: 10.3390/cancers13061293 (PMC7999522; doi:10.3390/cancers13061293)

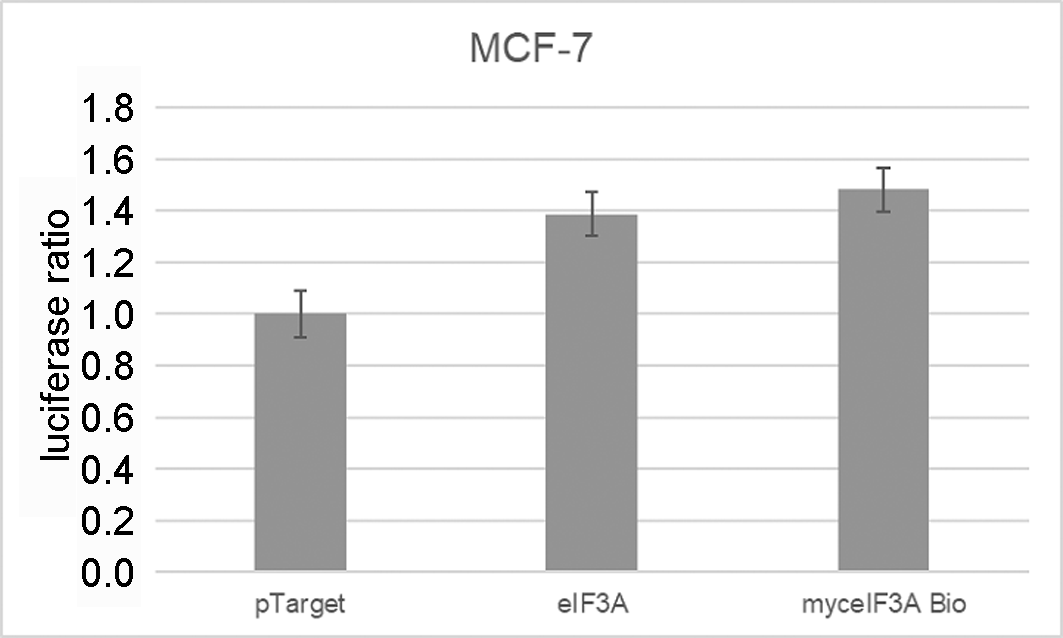

Supplement: Supplementary file 1 [file cancers-13-01293-s001.zip › sup fig S1.tif]

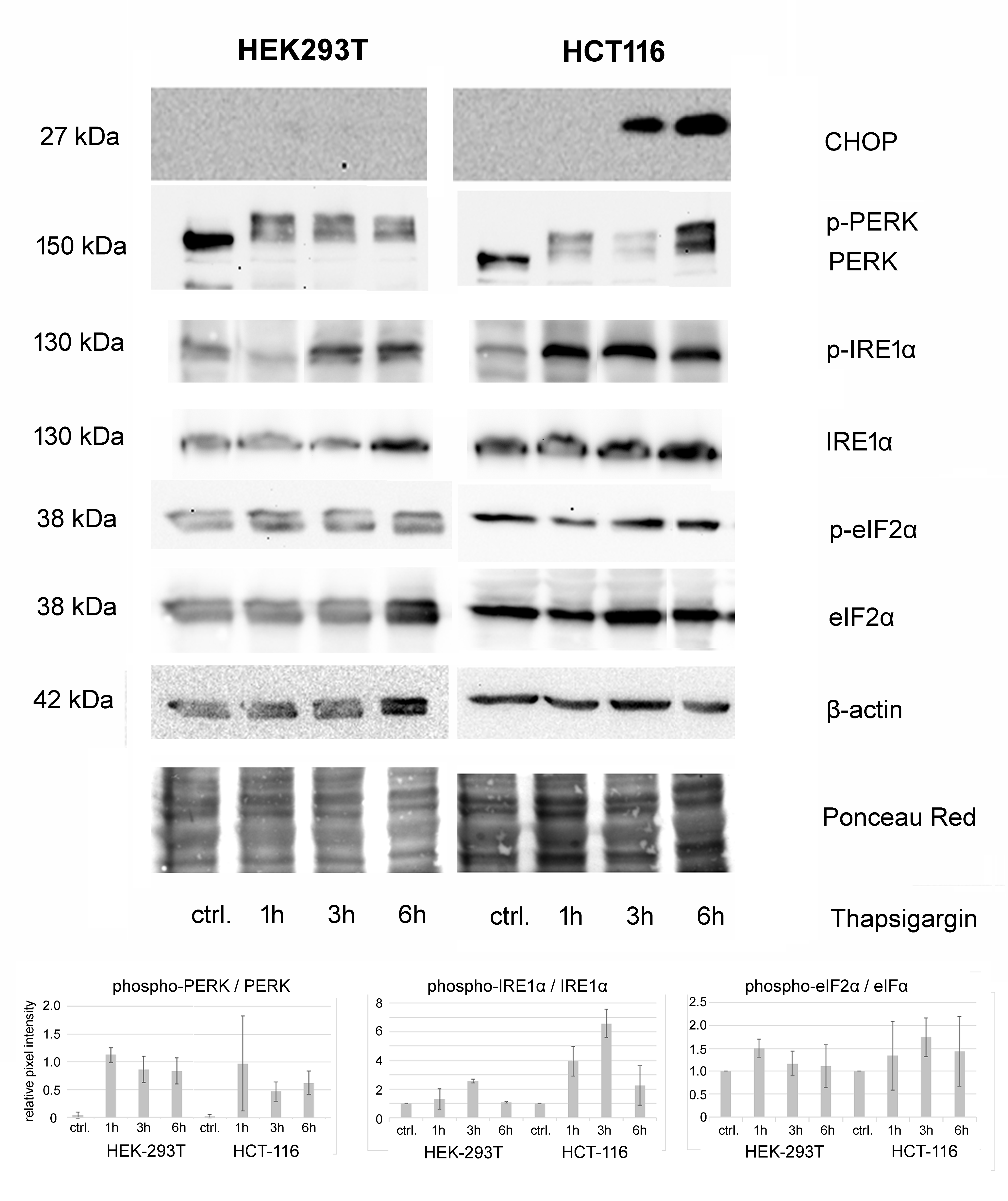

Supplement: Supplementary file 1 [file cancers-13-01293-s001.zip › sup Fig S2 western new copy 2.tif]

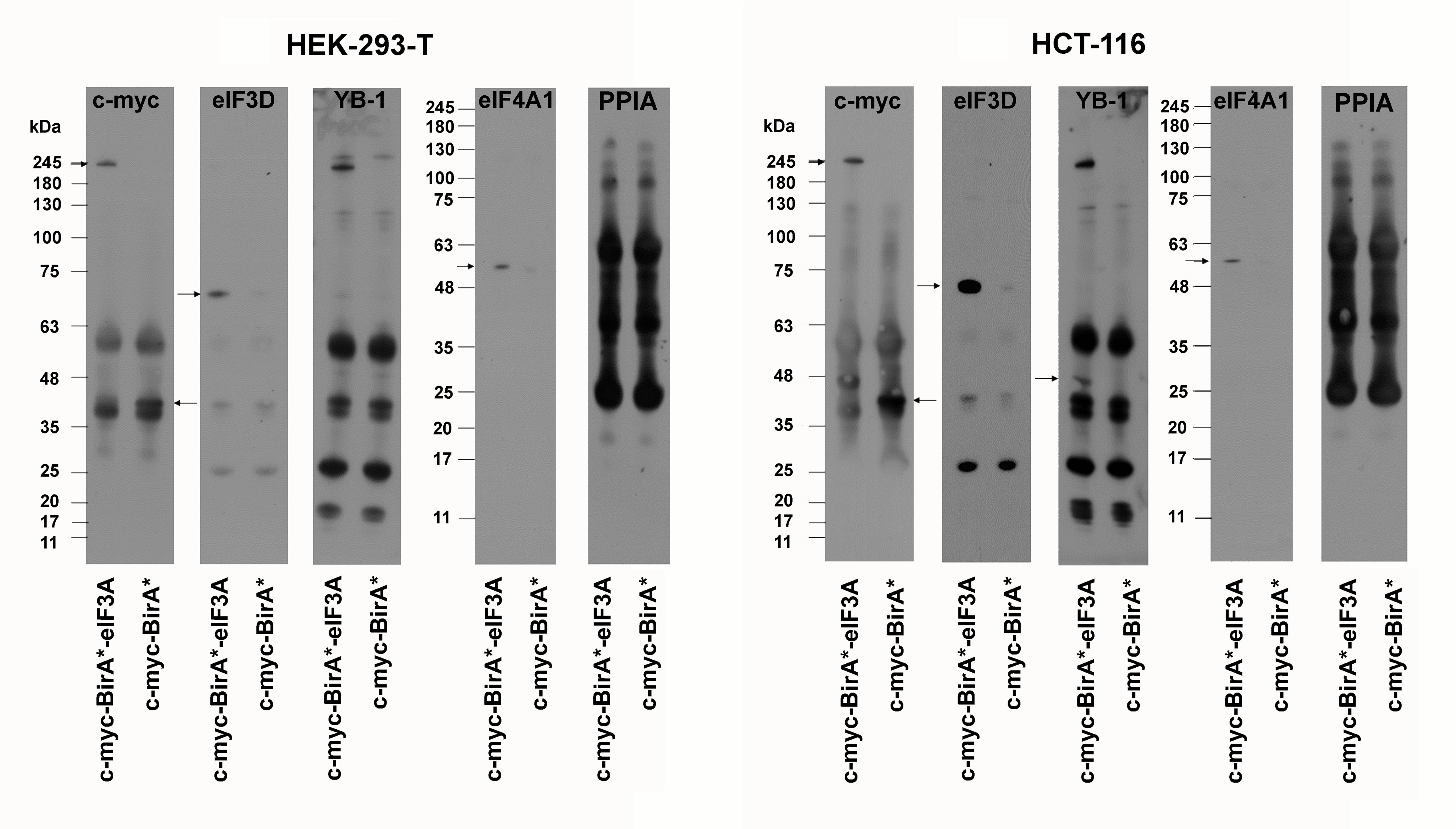

Supplement: Supplementary file 1 [file cancers-13-01293-s001.zip › Suppl fig 3 CoIP.tif]

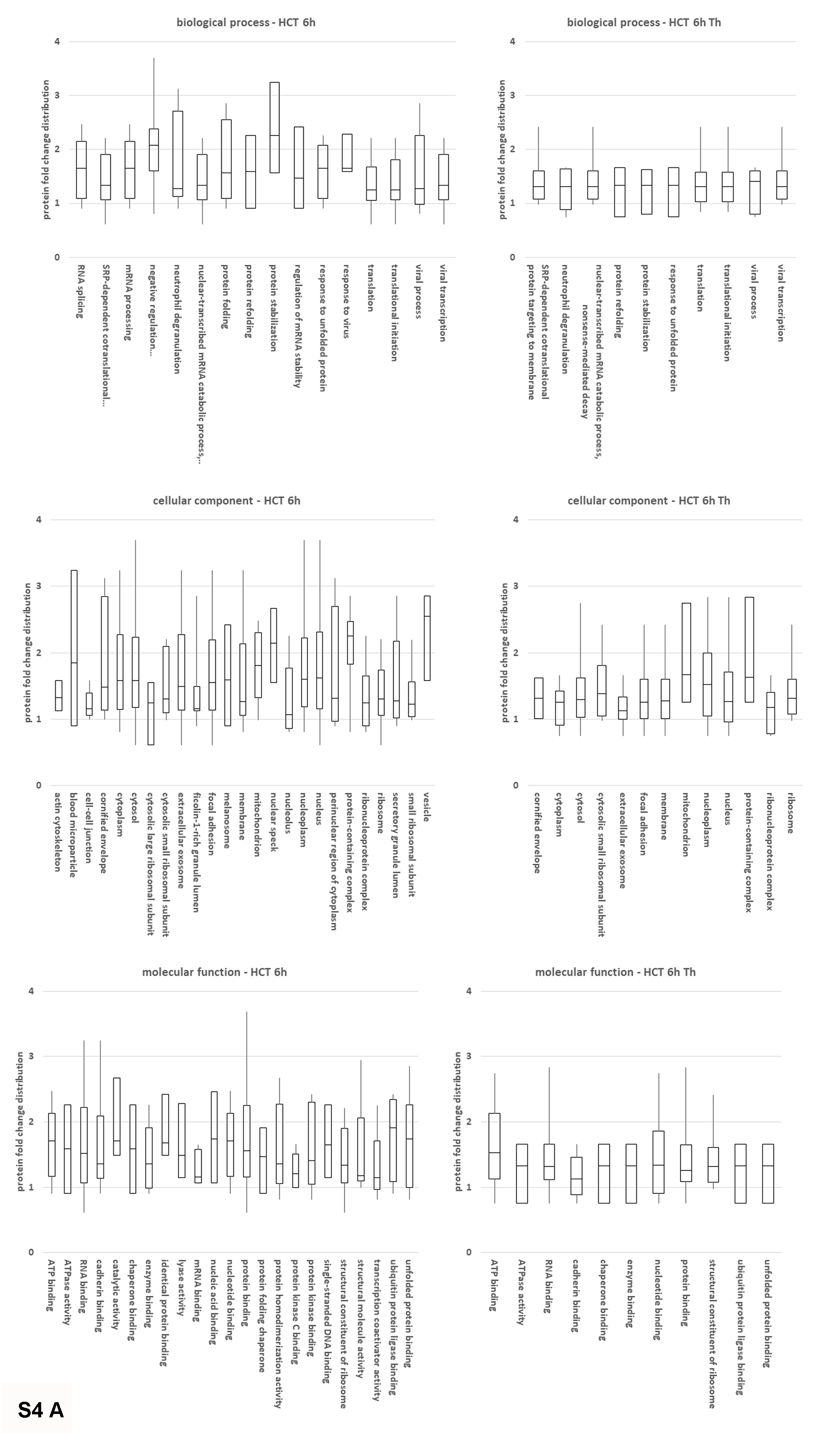

Supplement: Supplementary file 1 [file cancers-13-01293-s001.zip › Suppl Fig 4A HCT 6h.tif]

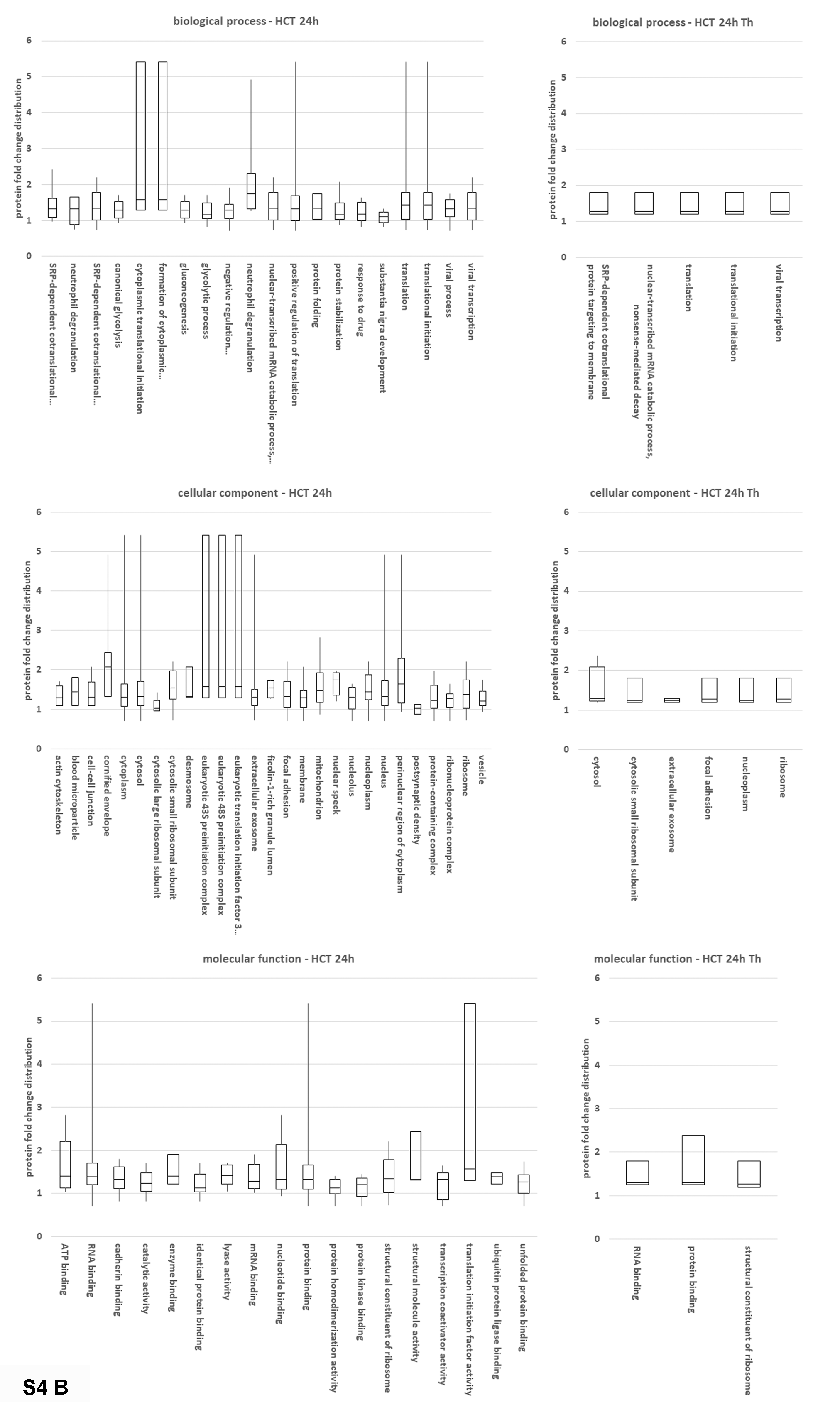

Supplement: Supplementary file 1 [file cancers-13-01293-s001.zip › Suppl Fig 4B HCT 24h.tif]

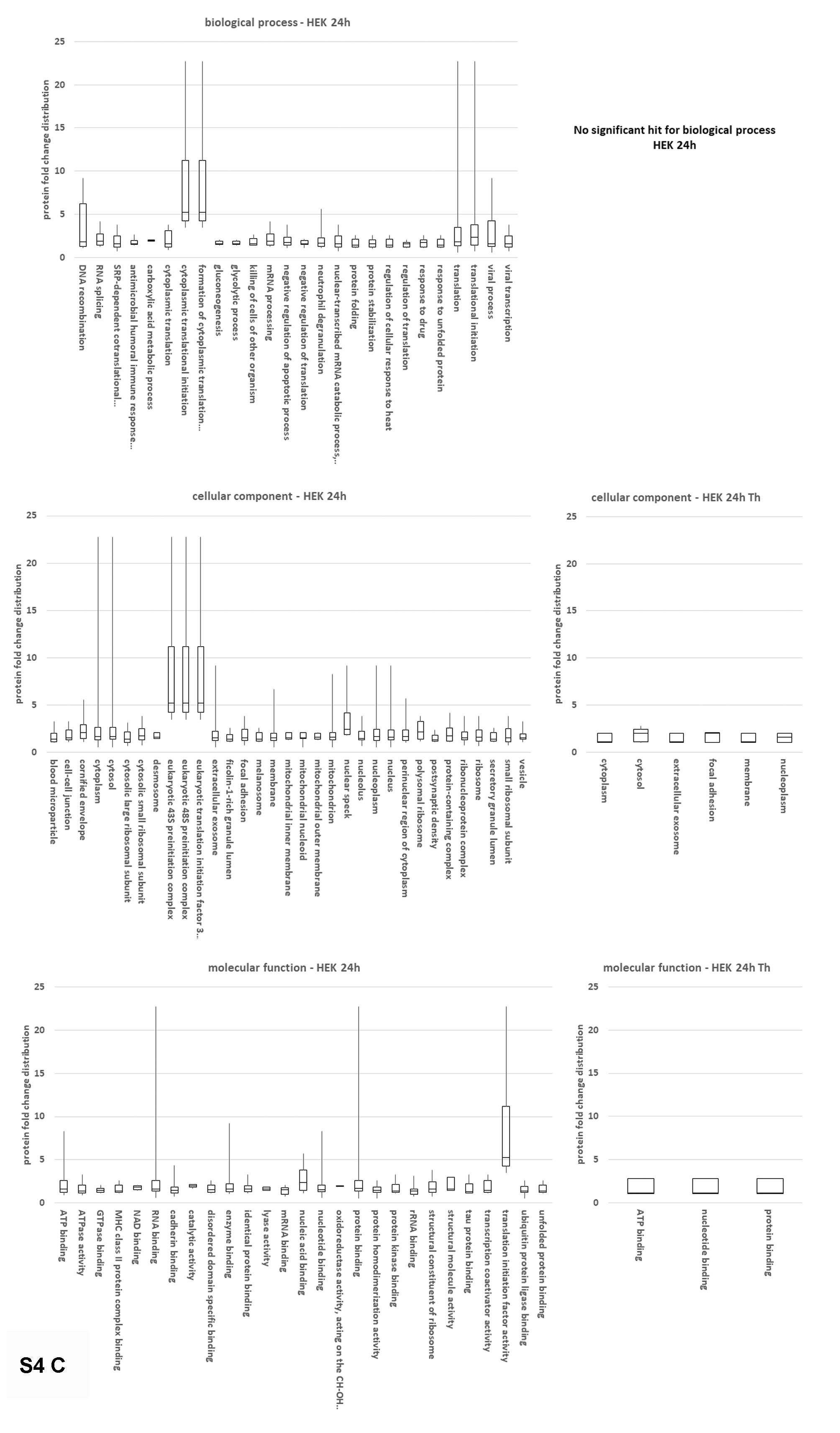

Supplement: Supplementary file 1 [file cancers-13-01293-s001.zip › Suppl Fig 4C HEK 24h.tif]

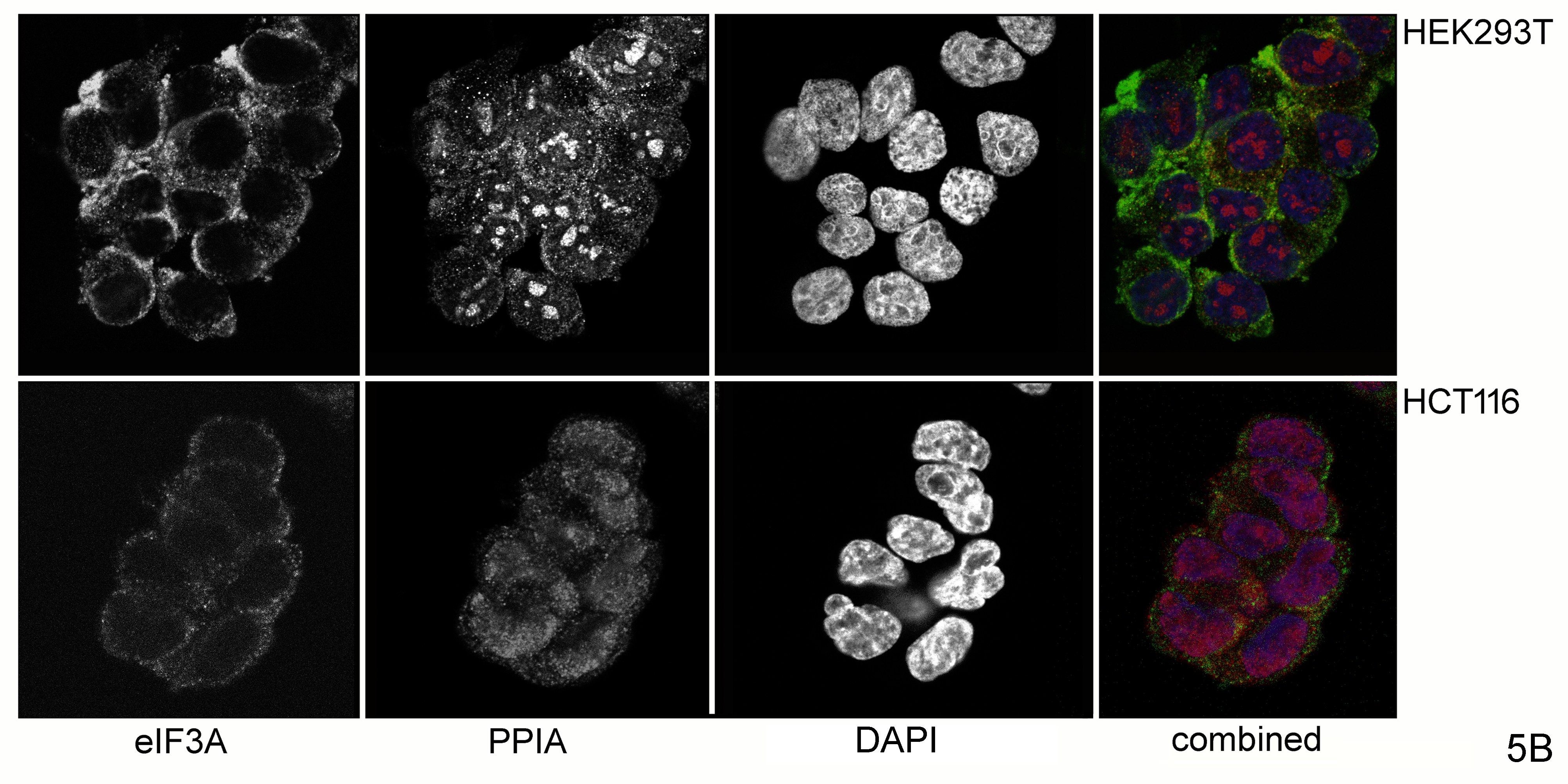

Supplement: Supplementary file 1 [file cancers-13-01293-s001.zip › Suppl Fig 5 B LSM cyclophilin eif3A_2.tif]

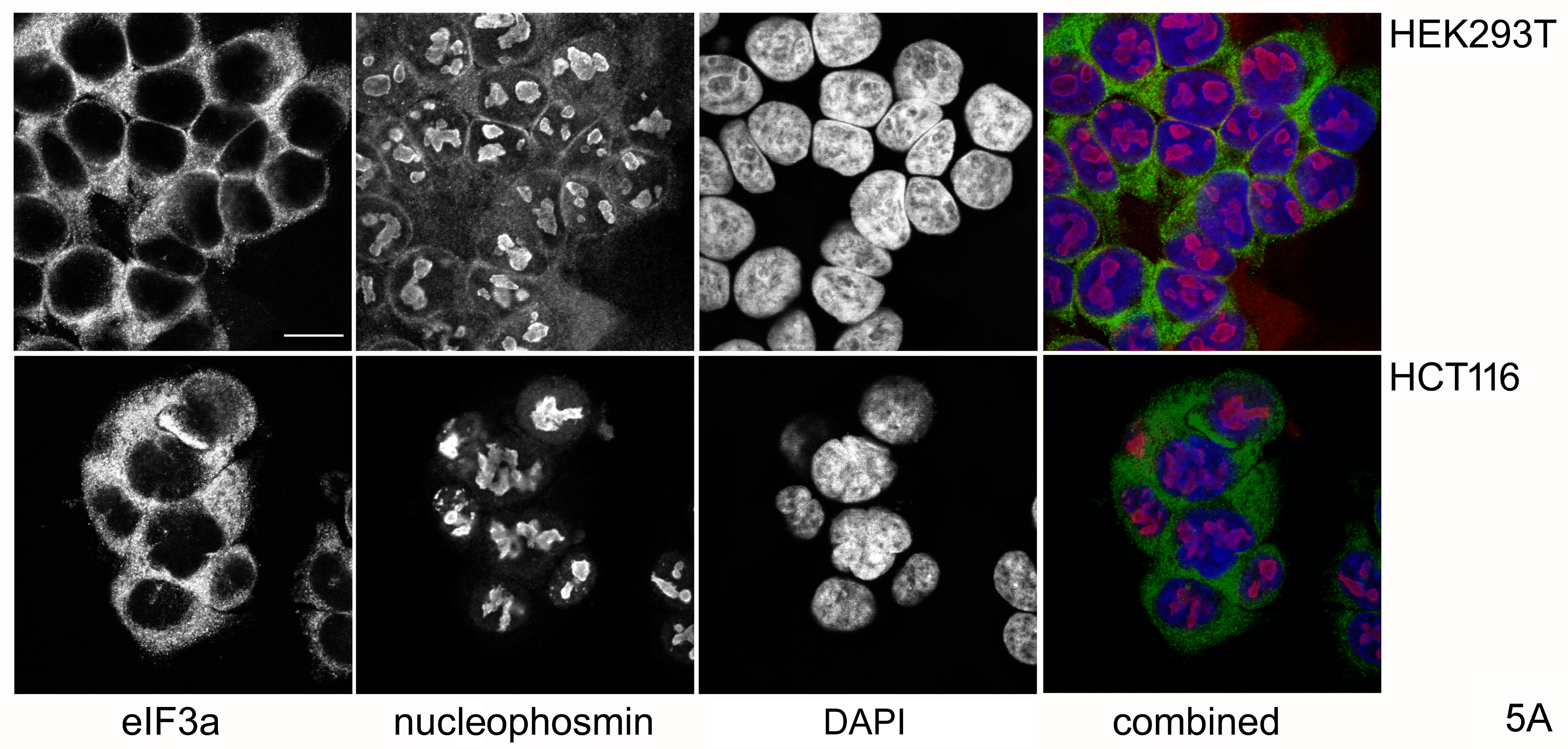

Supplement: Supplementary file 1 [file cancers-13-01293-s001.zip › Suppl Fig 5A LSM nucleophosmin eIF3a 600 dpi.tif]

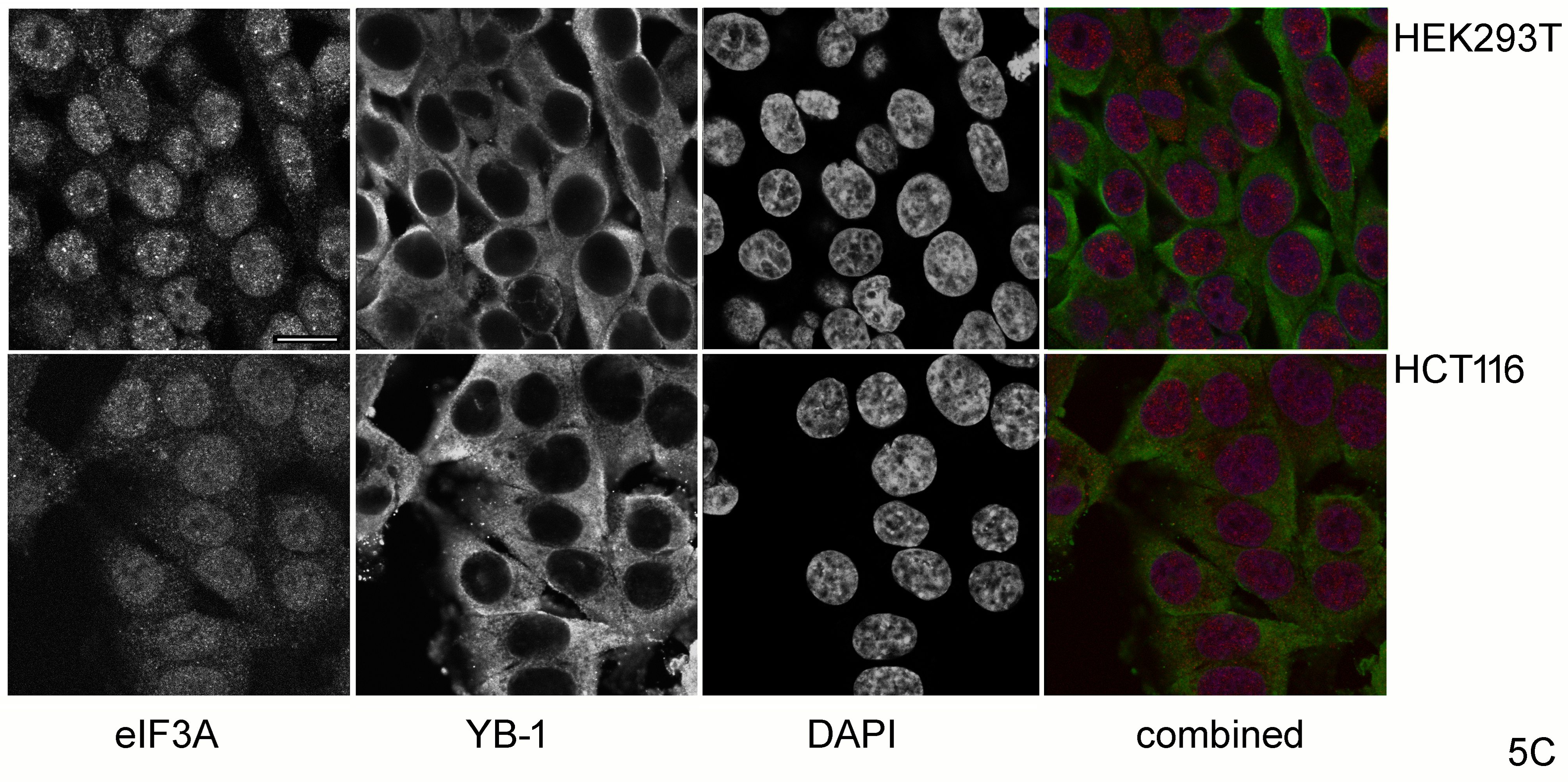

Supplement: Supplementary file 1 [file cancers-13-01293-s001.zip › Suppl Fig 5C YB1.tif]

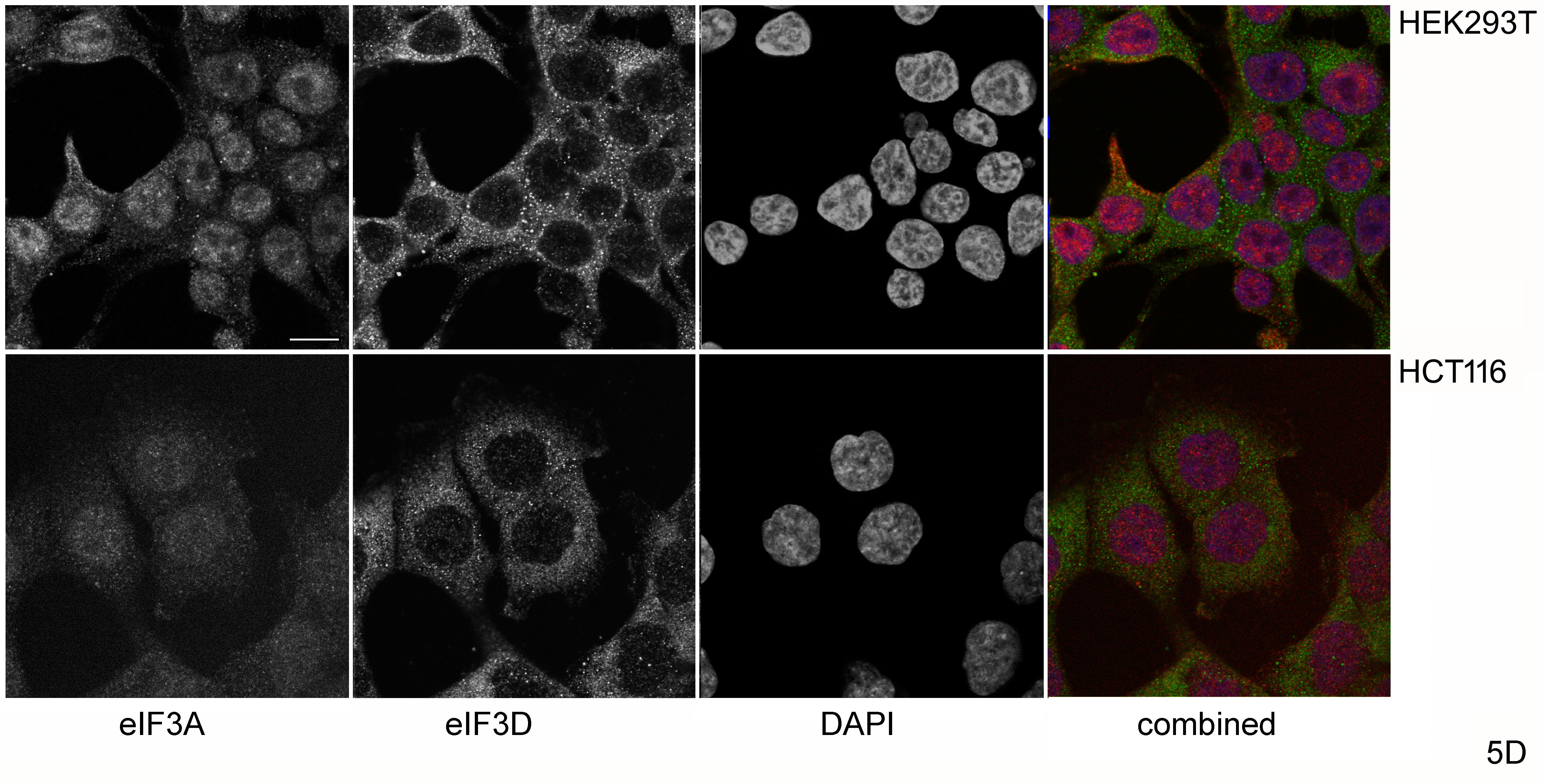

Supplement: Supplementary file 1 [file cancers-13-01293-s001.zip › Suppl Fig 5D 3D.tif]

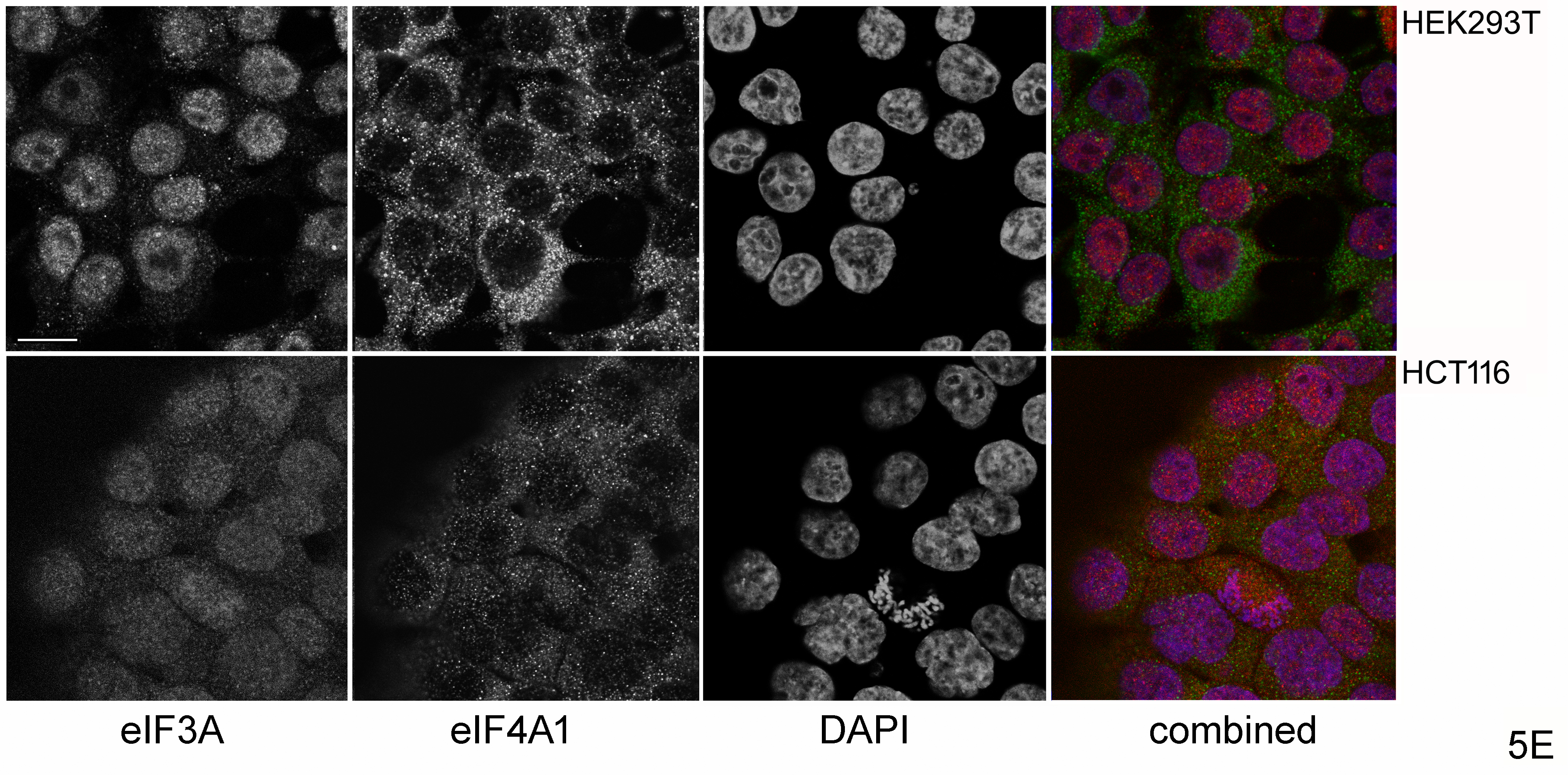

Supplement: Supplementary file 1 [file cancers-13-01293-s001.zip › Suppl Fig 5E 4A1.tif]
